# Rheology of Complex Topical Formulations: An Analytical Quality by Design Approach to Method Optimization and Validation

**DOI:** 10.3390/pharmaceutics15071810

**Published:** 2023-06-24

**Authors:** Lucas Chiarentin, Catarina Cardoso, Margarida Miranda, Carla Vitorino

**Affiliations:** 1 Faculty of Pharmacy, University of Coimbra, Pólo das Ciências da Saúde, Azinhaga de Santa Comba, 3000-548 Coimbra, Portugal; lczenith@gmail.com; 2 Laboratórios Basi Indústria Farmacêutica S.A., Parque Industrial Manuel Lourenço Ferreira, lote 15, 3450-232 Mortágua, Portugal; catarina.cardoso@basi.pt; 3 Coimbra Chemistry Centre, Institute of Molecular Sciences—IMS, Department of Chemistry, University of Coimbra, 3000-535 Coimbra, Portugal; 4 Egas Moniz Center for Interdisciplinary Research (CiiEM), Egas Moniz School of Health & Science, 2829-511 Caparica, Almada, Portugal

**Keywords:** rheology, analytical quality by design (AQbD), design of experiments (DoE), cream, method validation

## Abstract

Analytical method validation ensures that a method provides trustworthy information about a particular sample when applied in accordance with the predefined protocol. According to regulatory standards, the rheological characteristics of topically applied semisolid formulations are one of the key elements involved in microstructure equivalence documentation. Therefore, for generic drug product manufacturers, it is a dire need to take a step forward in rheology method development and validation procedures. This paper aims to apply Analytical Quality by Design (AQbD) principles towards the development and validation of rheology methods for topical creams, as complex semisolid formulations. Risk assessment was carried out through an Ishikawa diagram and an estimate failure mode, effects, and criticality analysis (FMECA). Sample application, peltier temperature control, and sample rest time were identified as critical method variables (CMVs), and a 2^3^ full factorial design was applied to understand their impact on rotational, creep recovery and, oscillatory measurements. The development of the method was carried out as per the ICH Q8-Q10, and Q14 guidelines and validated according to ICH Q2 (R2) guideline. The method demonstrated adequate precision (RSD < 15%), as well as selectivity. AQbD provided a comprehensive framework for developing a reliable and effective rheology method for this type of formulation.

## 1. Introduction

Topical products, commonly developed to exert a local action, have been used throughout history for cosmetic and therapeutic purposes [1]. Semisolid dosage forms aiming toward medical application, such as ointments, pastes, gels, rigid foams, and creams, display a complex multiphasic structure, which is deeply characterized by pseudoplastic behavior [2,3]. Semisolid topical formulations display interdependent relationships between their structure, physical properties, manufacturing process, and performance when compared to other dosage forms [4]. From a thermodynamic point of view, cream formulations are inherently unstable systems and, therefore, tend to break down over time due to the contribution of several physicochemical mechanisms, including gravitational separation, flocculation, coalescence, particle coalescence, Ostwald ripening, and phase separation [5]. As a result of these processes, changes in pH, viscosity, and color are frequently observed, which may compromise their stability and performance [6].

Clobetasol propionate (CP) is a prednisolone derivative that is commercially available in a wide range of topically applied dosage forms such as creams, ointments, solutions, foams, and gels [7]. CP applicability is closely related to its potency, being useful in a variety of skin disorders, ranging from itching, redness, dryness, crushing, scaling, inflammation, and discomfort of various scalp and skin conditions, including eczema and psoriasis [8,9,10,11,12,13]. Nevertheless, clinically, CP exhibits poor skin permeability, which leads to a reduction in the therapeutic efficacy at the target site. However, this drawback can be overcome by a proper selection of the right formulation, where the assessment of the microstructure presents itself as one of the features with paramount relevance in formulation performance. Taking this information into account, in the present study, a clobetasol propionate formulation was used.

The monitoring of rheological properties, by establishing the correlation between viscosity and shear stress, regards an important tool during the development of semisolid dosage forms since it sheds light on why some formulations flow, while others retain structure under shear. This behavior is of paramount importance from a patient compliance perspective [14,15]. On the other hand, the importance of these relationships is also crucial during the production stage, where rheological properties need to be assessed after manufacture and during shelf life in order to ensure that the formulation is physically stable [16]. The time- and temperature-dependent change in viscosity provides pharmaceutical formulations with rheological flexibility. This can subsequently affect the release profile of the active pharmaceutical ingredient from the semisolid matrix [2,17] and impact their permeation behavior [18].

Recently, the Food and Drug Administration (FDA) introduced the draft guideline on Physicochemical and Structure (Q3) Characterization of Topical Drug Products Submitted in ANDAs (2022) [19]. Furthermore, the European Medicine Agency (EMA) has also been vocal on this subject, through the release of the draft guideline on quality and equivalence of topical products (2018) [20,21]. In these documents, the characterization of rheological behavior is actively highlighted, as the applicants are highly encouraged to submit a complete rheological profile, addressing rotational and oscillatory measurements. For all the appointed reasons, the establishment of a well-defined and robust framework applied to rheology method development and validation is of outmost importance [15].

The Analytical Quality by Design (AQbD) concept, introduced in 2018, regards the translation of Quality by Design (QbD) principles to analytical method development [22]. The main rationale of AQbD relies on the continuous effort to improve analytical method selectivity and robustness, through a thoughtful identification and control of the critical method variables (CMVs) of the selected method [23,24]. The application of design of experiments (DoE) tools within this scope enables the attainment of mathematical relationships describing the impact of the CMVs on critical analytical attributes (CAAs) [25]. The interpretation of these relationships is crucial to define the optimal method conditions [26,27,28,29].

Taking into account the updated regulatory background, the specific objective of this work is to propose a workflow, based on AQbD principles, towards the development and validation of a rheology method applied to semisolid formulations. To the extent of our knowledge, this is the first literature report addressing this framework.

The following stages were considered within this scope:

- Definition of the analytical target profile (ATP): type of sample, type of the product, method application, type of analytical method, and instrument desirability;
- Risk assessment performance: made through an Ishikawa diagram and a failure mode, effects, and criticality (FMECA) analysis, in order to clearly define the selection of both CMVs and CAAs;
- Design of experiments (DoE): resorting to a 2*^k^* full factorial design to identify the parameters that have a more preponderant role in the method ATP, estimated through the desirability function;
- The last step comprised the performance of validation studies, a crucial part in every AQbD application. The optimized rheological settings were carefully validated in terms of precision and selectivity, in line with the existing guidelines, as well as other scientific reports [15,30,31,32,33].

**Figure 1:**
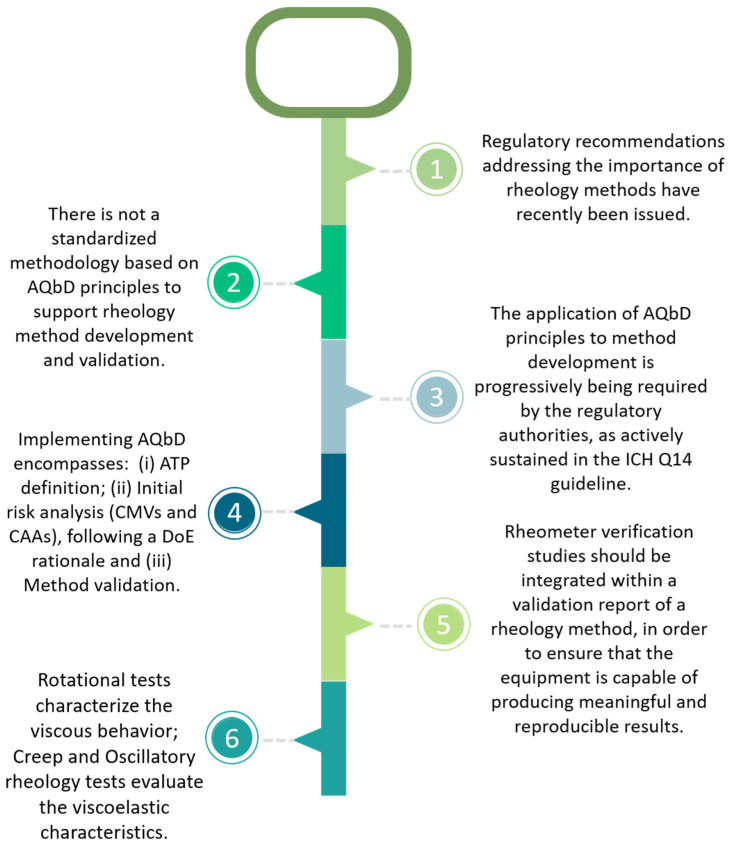
AQbD key concepts applied to a rheology method.

In an attempt to summarize the main objectives of the present work, as well as to pinpoint the key concepts supporting an AQbD-based development and validation approach addressing rheology methods, Figure 1 is introduced.

## 2. Materials and Methods

### 2.1. Materials

Clobetasol propionate, chlorocresol, glyceryl stearate, cetostearyl alcohol, citric acid, sodium citrate, propylene glycol, beeswax, and purified water were provided by Laboratórios Basi Indústria Farmacêutica S.A. (Mortágua, Portugal). Three batches of a commercially available clobetasol propionate 0.525 mg/g cream were used during rheology method applicability studies.

Viscosity reference standard RT5000 (Fungilab, Barcelona, Spain) was used for rheometer equipment verification studies.

### 2.2. Methods

The rheological analysis was carried out in a HAAKE^TM^ MARS^TM^ 60 Rheometer (ThermoFisher Scientific, Karlsruhe, Germany) with controlled temperature maintained by a thermostatic circulator and peltier temperature module (TM-PE-P) for cones and plates. All data were analyzed with HAAKE Rheowin^®^ Data Manager v.4.82.0002 software (ThermoFisher Scientific, Karlsruhe, Germany). Statistical analysis was performed using JMP v.17 software (Cary, IL, USA).

Viscosity measurements were also performed using a Rotavisc Lo-vi viscosimeter (IKA^®^, Werke GmnH & Co. KG, Mindelheim, Germany) with SP12 spindle at 1 rpm. These measurements were performed at 20 °C.

#### 2.2.1. Preparation of Clobetasol Propionate Cream Formulations

Clobetasol propionate o/w cream formulations were conventionally prepared using Ultra-Turrax X 10/25 (Ystral GmbH, Dottingen, Germany) equipment (Table 1). Both continuous and dispersed phases were separately prepared and heated to 60 °C. Afterward, the active pharmaceutical ingredient was solubilized in the dispersed phase. The produced cream formulations were stored at 20–25 °C. Batches of 0.5 kg were considered.

To document the discriminatory power of the proposed method, during validation studies, three formulations (Table 2) were manufactured:

(i) Formulation F1, considered as the main formulation and also used for DoE studies;
(ii) Formulation F2, containing a different glycerol monostearate content, while formulation variable. This excipient was selected due to its thickening role;
(iii) Formulation F3, which was produced using a different homogenization rate, while process variable. Product development studies revealed that a change in this operational setting highly impacted the rheological characteristics of the product.

Note that the latter two formulations (F2 and F3) were considered to establish the rheology method selectivity.

#### 2.2.2. Equipment Verification

A viscosity curve was traced using the reference standard to verify the rheometer. Two temperatures were considered, 25 °C to mimic standard manufacturing specifications and 32 °C to mimic skin application. Triplicate measurements were performed.

#### 2.2.3. AQbD Rheology Method Development

AQbD is rooted in ICH guidelines Q8 and Q9, which have been translated into the analytical domain through several white papers as well as the USP <1220> [34], Simões and coworkers [15], and forthcoming ICH Q14 [22]. The philosophy behind and strategies for the implementation of AQbD and the associated life cycle management of an analytical method have been combined according to the recently issued FDA draft guideline on Physicochemical and Structure (Q3) Characterization of Topical Drug Products Submitted in ANDAs (2022) [19] and the EMA draft guideline on quality and equivalence of topical products (2018) [20,21].

##### Analytical Target Profile

ATP refers to a prospective summary of the quality characteristics intended for the analytical method. In other words, it describes the appropriate attributes to be measured and relevant performance characteristics for a specific analytical method [27,35]. In this context, a comprehensive review of the ATP points to the selection of the critical method variables, method design, and development activities [36].

The ATP, described in Section 3.2, was tailored taking into account the Product Quality Target Profile (QTPP) and Critical Quality Attributes (CQA). Furthermore, regulatory requirements as well as relevant guidelines were likewise considered within this scope [22].

##### Initial Risk Assessment

Risk assessment regards the identification of the analytical parameters that could negatively impact CAAs. This assessment helps to identify the inherent risks and, at the same time, provide measures, processes, and controls to reduce their impact [37,38]. An Ishikawa diagram was traced to identify the risks, in order to provide a basis for risk evaluation and decisions on risk control. Afterward, the risk evaluation was performed by means of a failure mode, effects, and criticality analysis (FMECA), with the sole purpose to increase the knowledge of risk and to prevent failure. The output of an FMECA regards a relative risk “score” for each failure mode, which is then used to rank the modes on a relative basis. Risk quantification is to be performed by considering the severity (S), probability of occurrence (O), and detectability (D) of each parameter using a numerical scale 1–5, with 1 being the lowest severity, probability, and undetectability, and 5 the highest. For each factor, the rank and prioritization of the risk were conducted according to the risk priority number (RPN) given by RPN = S × O × D. The factors presenting higher RPN values were subjected to a further optimization analytical process [39,40,41].

##### Method Optimization

After risk assessment, design of experiments (DoE) should be conducted for method development, in order to screen or optimize method conditions as highlighted per Fukuda and collaborators (2018) [25].

The choice of suitable CMVs is extremely important, as it conditions the experimental results and respective interpretation. These were considered for rotational, creep recovery, and oscillatory measurements. This approach aimed at assessing the impact of different rheological critical method variables. The selected CMVs included the sample application mode, peltier temperature control, and sample rest time.

For DoE studies, a two-level full factorial design, 2*^k^*, with three variables was used. *K* factors were considered, each at 2 levels, including low and high levels. These levels are numerically expressed as −1, and +1, respectively. By applying a 2^3^ full factorial design, a total of eight autonomous experiments (three replicates per experiment) were conducted to determine the impact of the selected CMVs on the responses.

To evaluate the DoE responses, both Student’s *t*-test and ANOVA were conducted to assess the statistical significance of the experimental parameters in the regression model. Please note the formulation F1 was used for DoE studies.

1. Rotational measurements

Rotational measurements were performed using a cone (P35 2°/Ti, 35 mm diameter, 2° angle)-and-plate (TMP 35) geometry configuration. The measurements were carried out using a gap distance of 1 mm.

Viscosity curve

The viscosity curve (*η* = f(γ)) exhibits a dependence of both shear stress (*τ*) and apparent viscosity on the shear rate (ẏ). Furthermore, the viscosity curve is also time-dependent when considering a controlled-rate mode [42,43]. To trace the viscosity curve, the shear rate was linearly (CS mode) increased from 5 to 500 Pa for DoE studies, whilst for method validation, a 10 to 900 Pa range was considered. Both methods regarded a run time of 300 s and the collection of 30 data points.

The following CAAs were regarded for this test: zero-shear viscosity (*η*_0_), yield point (*τ*_0.ROT_), and infinite-shear viscosity (*η_∞_*).

Thixotropic profile

Flow curves (*τ* = f(γ)) were attained by the shear rate ramp-up from 0.1 to 300 s^−1^ (ascendant curve) and ramp-down from 300 to 0.1 s^−1^ (descendent curve). The thixotropic behavior was estimated by considering the hysteresis loop area (S_R_).

2. Creep recovery

The creep recovery test aims to describe the slow steady flow of a material under low-stress conditions [44]. More specifically, it evaluates the elastic and viscous components of the samples and their recovery profile, after being subjected to a shear stress. The test must be performed within the viscoelastic region, where the microstructure remains undisturbed. The measured response in a creep test is usually presented in terms of creep equilibrium compliance (J_e_, Pa^−1^), which corresponds to the ratio of the measured strain to the applied stress, or inverse modulus and the response elastic reformation (γ_e_, %) [45,46,47]. The creep recovery test was performed using a cone (P35 2°/Ti, 35 mm diameter, 2° angle)-and-plate (TMP 35) geometry configuration. The measurements were carried out using a gap distance of 1 mm, with a shear stress of 50 Pa (within the LVR), over 200 s on the sample, followed by a recovery phase where the stress was suddenly removed, and the sample was allowed 200 s to recover the elastic part of the deformation.

3. Oscillatory measurements

Oscillatory measurements were performed using a plate (P35/Ti, 35 mm diameter)–plate (TMP 35) geometry configuration. The measurements were carried out using a gap distance of 1 mm.

Amplitude sweep

The oscillatory stress sweep test was performed at a constant frequency of 1 Hz from 0.5 to 1500 Pa. The following CAAs were regarded for this test: linear viscoelastic region (LVR), yield point (τ_0.OSC_), and flow point (τ_f_).

Frequency sweep

The frequency sweep was conducted from 70 to 0.1 Hz, at a constant shear stress of 5.0 Pa. The following CAAs were regarded for this test: elastic modulus (G′) and viscous modulus (G″).

##### Definition of the Optimal Operational Settings

In an attempt to determine the optimal operational settings, the responses were ranked according to their impact on the rheology behavior, as well as meeting the ATP criteria for the analytical procedure [48].

#### 2.2.4. Method Validation

After DoE experiments, the best conditions proceeded for validation studies. Since there are no specific guidelines for rheology method validation, the ICH Q2 (R2) guideline as well as the rheology tutorial proposed by Simões et al. (2020) were regarded as directives for addressing method precision and selectivity [15,22,49].

##### Precision

The precision of an analytical method procedure expresses the closeness of agreement between a series of measurements obtained from multiple samplings of the homogeneous sample, applied under the prescribed conditions [49]. Rheology method precision was determined by assessing the method repeatability and intermediate precision by F1 formulation. The acceptance criterion was set to an % RSD less than 15% [50]. A minimum of twelve determinations for each measurement were considered.

##### Selectivity

The selectivity of the rheology method refers to the ability of the method to detect changes in product performance, generally demonstrated by determining the effect of deliberate meaningful changes in the formulations [26]. In other words, the selectivity regards the ability of the method to provide a different response to a different formulation. To achieve this, two different formulations with changes in critical manufacturing variables and quantitative excipient composition (F2 and F3, previously detailed in Section 2.2.1) were specifically manufactured and cross-compared with the nominal formulation (F1). Then, the method selectivity was documented statistically by ANOVA with a Tukey multiple comparison test. Pairwise comparisons between the nominal formulation (F1) and the specifically manufactured formulations F2 and F3 were conducted. The differences between the means were considered significant at a value of *p* < 0.05.

#### 2.2.5. Statistical Analysis

Statistical analysis was performed using GraphPad Prism 8 software (San Diego, CA, USA) by applying a one-way ANOVA with a Tukey multiple comparison test. JMP v.17 software (Cary, IL, USA) was used for statistical analysis of the fitted models, including Student’s *t*-test, in order to test whether the terms were statistically significant in the regression model. The statistical analysis was considered significant when the regression Prob > F and *t*-test Prob > |t| were less than 0.05. The maximum squared regression coefficient (R^2^) indicated how well the model fitted to the experimental data, and the closer the value is to 1, the better the fit.

## 3. Results and Discussion

As described in the introduction section, several steps were considered to reach the ultimate goal of the present work: the application of an AQbD framework to the development and validation of a rheology method.

**Figure 2:**
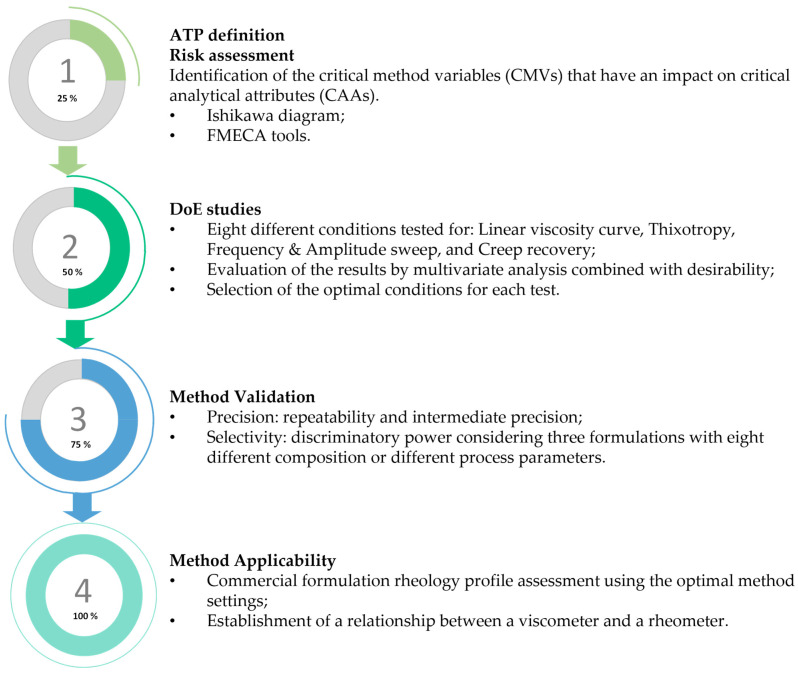
Workflow followed for the development, validation, and application of the rheology method.

The initial and more theoretical components of the present work contemplated the definition of the ATP, as well as a complete risk assessment analysis resorting to Ishikawa and FMECA tools. DoE studies were then performed for all rheology methods considered, taking into account the CMVs chosen—sample application mode, peltier temperature control, and sample rest time. A total of eight autonomous experiences with three replicates were considered for each method. These results were analyzed using Student’s *t*-test, ANOVA, and the desirability function to determine the optimal conditions for each method. In the last stage of the work, method validation and method applicability studies were conducted. Figure 2 summarizes the workflow followed in the present work.

### 3.1. Equipment Verification

In order to comply with good manufacturing practices (GMPs), manufacturers should have a rigorous verification or qualification policy for all software systems as well as equipment used during production and quality control operations. Equipment verification was performed with a peltier controlled temperature at 32 °C. This temperature was chosen to mimic the physiological skin temperature [33,51,52]. Environmental factors such as a suitable working area, workbench levelness, and a satisfactory compressor system were ensured.

The acceptance criterium (RSD < 15%), which is in agreement with FDA guidelines, was considered.

Table 3 depicts the viscosity results retrieved from the Newtonian reference standard.

### 3.2. AQbD Rheology Method Development

#### 3.2.1. Definition of ATP

The ATP of the rheology method applied to the clobetasol propionate cream formulations is depicted in Table 4. An effort was made to standardize the rheological methodology to comprehensively address the characterization of all operational parameters in a robust and efficient manner.

#### 3.2.2. Initial Risk Assessment

In order to mitigate the initial risk assessment, an Ishikawa diagram and FMECA were carried out to identify and quantify all possible causes of disruption during rotational, creep recovery, and oscillatory measurements.

**Figure 3:**
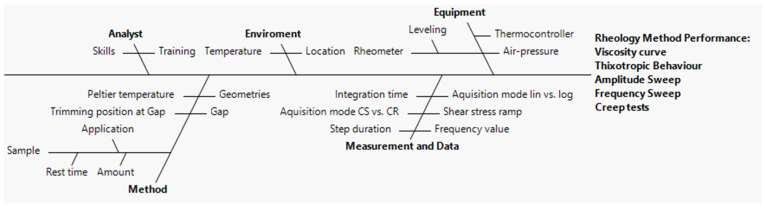
Ishikawa diagram depicting the cause-and-effect relationship on the selected CAAs of the rheology method.

An Ishikawa diagram (depicted in Figure 3) dissects the method development process into various fractions such as analyst, environment, equipment, method, measurement, and data [53]. Each fraction provides an insight into factors that can affect the CAAs. As shown in Figure 3, according to previous knowledge, it was possible to identify several analytical settings which may have a direct repercussion on the rheological output. Based on this analysis, FMECA was carried out in order to rank the risk.

FMECA is a tool to identify potential problems during method development. It is an inductive method used for identification of hazards of a system with single-point failure. Table 5 shows the criteria used to assess FMECA scores. Risk acceptance is achieved by comparing the RPN score with defined acceptance levels. FMECA aids to rank and prioritize these factors into low, medium, and high risks based on analytical method hazards, as well as the probability that it will occur [54,55,56,57,58].

As previously mentioned, several CAAs were retrieved from each rheological test, according to Table 6. These are described below.

For rotational measurements, two tests were considered: viscosity curve and thixotropy tests. The following responses were studied for the viscosity curve:

- Zero-shear viscosity is the limiting value of the shear rate-dependent viscosity function at an “infinitely low” shear rate, meaning the first Newtonian range with the *plateau* value;
- Infinite-shear viscosity is the limiting value of the shear rate-dependent viscosity function at an “infinitely high” shear rate, meaning the last Newtonian range with the *plateau* value;
- Yield point (also called yield stress) is the lowest shear stress value above which a material behaves as a fluid, and below which the material acts as a solid; in other words, it is the minimum shear stress required to initiate flow [59].

The time-dependent behavior, also known as thixotropic behavior, refers to the reduction in structural strength during a shear load phase and a more or less rapid but complete structural regeneration during the subsequent period of rest. The area between the upward and downward curves is called the “hysteresis area”; if the value is positive, the sample shows structural breakdown and if the value is negative, the sample shows structural build-up upon shearing. The hysteresis area indicates how fast the sample structure recovers after the load is removed [59].

Oscillatory tests were likewise performed. These were divided into two main parts: amplitude and frequency sweep experiments. The responses for amplitude sweep were:

- Linear viscoelastic range (LVR region), which indicates the range in which the test can be carried out without destroying the structure of the sample and represents a material’s ability in preserving its microstructure when exposed to rising shear stress;
- Yield point, which stands for the stress value at which the curve begins to deviate noticeably from the LVR *plateau* or from the corresponding fitted straight line used for analysis;
- Flow point, representing the shear stress value where the moduli cross over (G′ = G″) [41].

Regarding frequency sweep, the storage modulus (G′) represents the magnitude of energy stored in a material, whereas the loss modulus (G″) represents the energy loss due to viscous dissipation. Therefore, a material presents elastic properties when G′ < G″ and viscous properties when G′ > G″ [60].

Because of the relatively low consistency of many pharmaceutical formulations, it is often difficult to apply small enough stresses within the linear viscoelastic region for an oscillation test. In this context, the evaluation of the creep recovery test is an alternative for determining the relaxation time and viscoelastic properties of a material. A constant stress below yield stress is applied to the material and the deformation is monitored with time. Compliance (J) is defined as the reciprocal of the modulus, J = 1/G = γ/*τ*, where G is the modulus and γ is the strain. Creep recovery behavior aids in understanding the deformation mechanisms of the sample. Creep testing delivers strain or compliance measurements as a function of time under very slow stresses. High values of creep equilibrium compliance are characteristic of weaker internal structures [45,61,62]. Equilibrium compliance is the elastic response to strain of a viscoelastic material [63]. The elastic reformation value indicates the elastic portion of the viscoelastic behavior [59].

Table 7 shows the failure mode (the way in which a failure is observed), failure cause (the determination of causes of the failure mode), failure effect (the immediate consequences of a failure on the operation, function, or functionality), risk priority number (RPN), and recommended actions.

The effects can be further classified with the calculation of theRPN, which is based on three categories (RPN interval for each category: category 1: low risk, value < 20; category 2: medium risk, value between 20–30; category 3: high risk, value > 30). Acceptance levels must be defined on a case-by-case basis, always focused on method quality. According to prior knowledge, the following CMVs were considered and may pose a direct repercussion on rheological endpoints: sample application, sample rest time, and peltier temperature.

#### 3.2.3. Optimization of the Rheological Measurements

The optimal conditions for the rheological measurements were selected using a two-level, three-factor, 2*^k^* full factorial planning resorting to JMP 17.0 software (Cary, IL, USA). Most CAAs were identified based on the initial risk assessment analysis (Figure 3 and Table 7). Eight autonomous analyses, with three replicates each, were conducted to determine the effect of the three factors of each rheology endpoint (Table 8 and Table 9).

**Figure 4:**
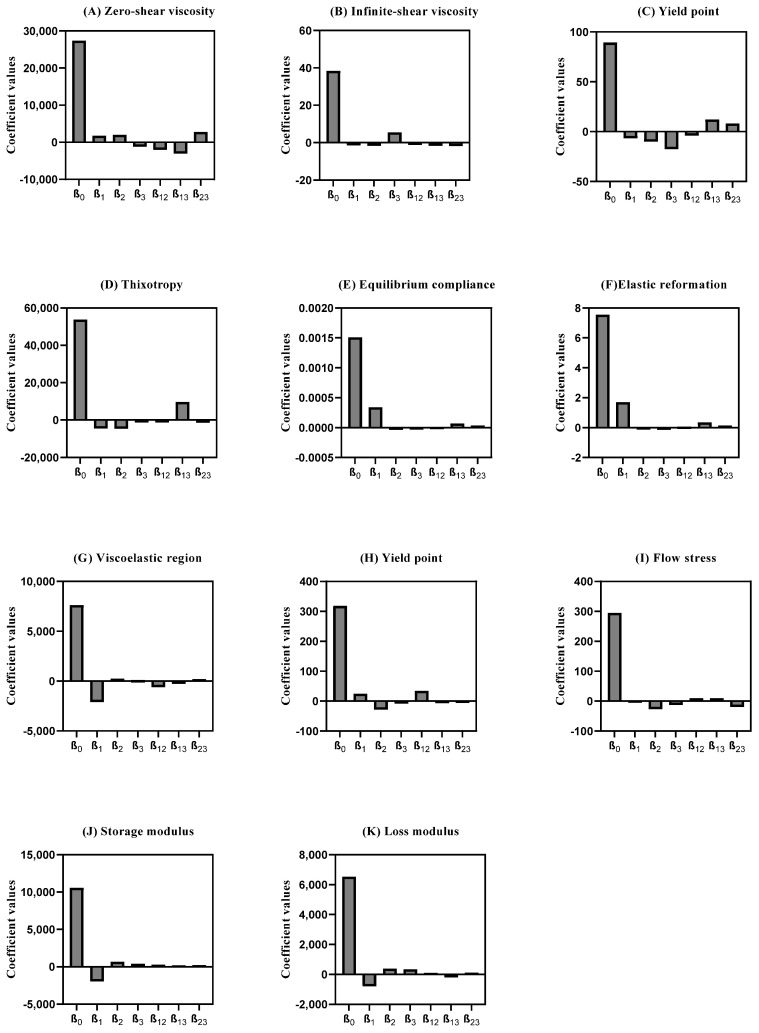
Coefficient values of rheology measurements were extracted from mathematical models obtained from DoE. (**A**) Zero-shear viscosity; (**B**) Infinite-shear viscosity; (**C**) Yield point (rot); (**D**) Thixotropy; (**E**) Equilibrium compliance; (**F**) Elastic Reformation; (**G**) Viscoelastic region; (**H**) Yield point (osc); (**I**) Flow stress; (**J**) Storage modulus; (**K**) Loss modulus. Results report to n = 3.

To assess the influence of each factor and their respective combination on the responses, the polynomial coefficients were determined for each rotational, creep recovery, and oscillatory measurement’s response. A higher coefficient magnitude indicates a stronger main effect on the system. Additionally, if the coefficient has a positive sign, an increase in its level leads to an increase in the response. If the sign is negative, an increase in the independent variable level leads to a decrease in the response [64,65]. The integrated analysis of these responses yielded distinct models, whose coefficient values are presented in Figure 4 and Tables S1 and S2. Further, these results are discussed in the following sections.

The main goal supporting method optimization relied on the maximization of most of the rheology outputs, thus enabling a more comprehensive documentation of the rheology behavior, without compromising the discriminatory capacity of the method [66].

Actual by predicted plots of rotational and creep responses (CAAs) presenting a better goodness of fit from DoE experiments are shown in Figure S1 and S2. The diagonal line corresponds to the Y = X line. For a theoretical perfect fit, all the points would be on this diagonal. These curves provide a visual indication of significance at the 5% level.

The desirability (D) function is described as the weighted geometric mean for several responses or, alternatively, a value between 0 and 1 per response. A value of D different from zero indicates that all responses are in a desirable range, whilst a value close to 1 is pointed out as the combination of the different criteria considered optimal (Figures S3 and S4). As such, when D = 1, it means that the response values are close to the target ones [65]. In addition to that, Tables S3 and S4 depict the evaluation of ANOVA also performed for model fitness.

**Figure 5:**
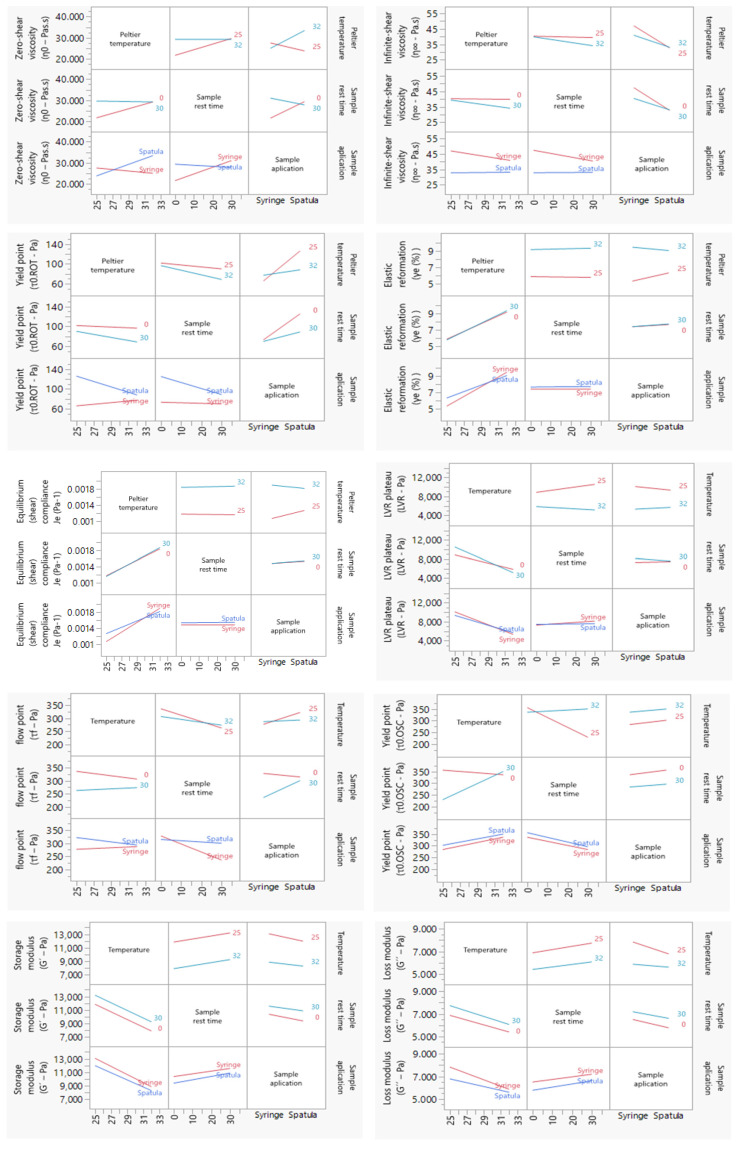
Interaction plots of the DoE rheology responses. A line segment is represented per level of the row effect, and response values predicted by the model are summarized by line segments. Note that non-parallel line segments give visual indication of possible interactions. However, the *p*-value for such a suggested interaction should be verified to consubstantiate that it exists; please report to Figure 5. Results report to n = 3.

Interaction plots (Figure 5) display means for the levels of one factor on the x axis and a separate line for each level of another factor. Interaction effects were also analyzed by regression analysis and ANOVA for the responses in rotational, creep recovery, and oscillatory measurements. When the effects were significant, the results were interpreted considering the interaction effects. Note that parallel lines indicate no interaction and intersecting lines indicate possible interactions.

After conducting the DoE experiments and analyzing data, with the response of the desirability function, it was possible to establish the final rotational, creep recovery, and oscillatory measurement conditions, which were then applied during method validation studies.

##### Rotational Measurements

Viscosity curve

**Figure 6:**
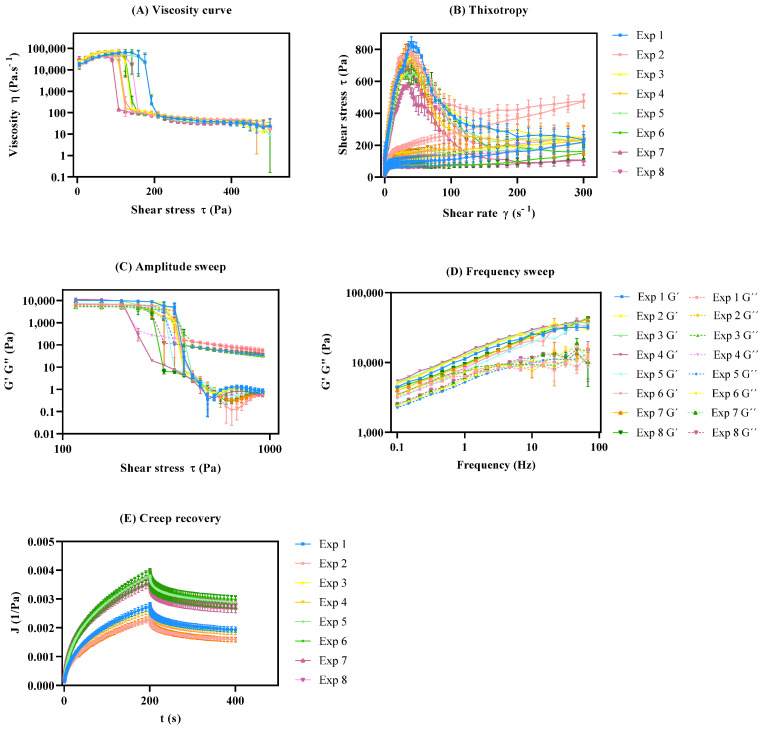
Rheology profiles of DoE. All results report to *n* = 3 ± SEM. (**A**) Viscosity curve; (**B**) Thixotropic behavior; (**C**) Amplitude sweep; (**D**) Frequency sweep; (**E**) Creep recovery.

As displayed in the viscosity curve (Figure 6A), all experiments exhibited a non-Newtonian behavior, since the viscosity decreased with an increase in the shear rate, which classifies the system as pseudoplastic or shear-thinning [67]. The zero-shear viscosity depicts the strain response in the low-stress region and yields a high-viscosity plateau labeled as the zero-shear viscosity (*η*_0_) [18,68]. This endpoint was determined at approximately 22.0 Pa and is useful, as it reflects product viscosity in the steady state or, in other words, the product’s state within the container. This rheological response is mostly affected by the interaction between the peltier temperature and sample application (*β*_13_). Another major effect of this specific rheological response relies on the synergy effect between the sample rest time and sample application (*β*_23_). This may occur because the syringe causes major extrusion in the sample prior to the analysis, since as the stress increases, plastic flow occurs at critical stress.

On the other hand, the infinite-shear viscosity (*η***_∞_**) presented a less expressive coefficient magnitude, when compared to the *η*_0_ (Figure 5B). This endpoint refers to the second constant viscosity plateau and can be several orders of magnitude lower than *η*_0_ depending on the degree of shear thinning [69,70,71]. The results demonstrated that the sample application had a positive impact on the response (*β*_3_), whilst sample application by means of a syringe tended to increase this response. Once again, the interaction between peltier temperature and sample application (*β*_13_) proved to have a significant, but negative, impact on this CAA.

Another rotational endpoint regards the yield point. This is the minimum force that must be applied to start sample flow, and was calculated from the “steady stress sweep” method [72]. DoE results show that the sample application is of outmost importance, with the syringe application yielding lower results. This rheological endpoint was also negatively affected by the sample rest time, with lower rest times leading to lower yield point values. The interaction between the peltier temperature and the sample application (*β*_13_) is also considerable, however it has the opposite trend [73].

The desirability profiler for the viscosity curve suggests performing the analysis at 32 °C for peltier temperature, zero minutes for sample rest time, and sample application using a spatula (Figure S2).

Rheological modeling

To obtain details of DoE rheological parametric evaluation, viscosity and shear rate rheograms were analyzed by fitting results with various models, such as Ostwald–de Waele, Cross, Herschel–Buckley, Bingham, and Casson models (Table 10).

The flow behavior of samples without yield stress (*τ*_0_) can be described using the Ostwald–de Waele Equation (1):

(1) $$\eta =k\;x\;{ẏ}^{n-1}$$

where *η* is the viscosity (Pa·s^−1^), *k* is the flow coefficient, and exponent *n* refers to the flow index. It indicates the following: *n* < 1 for shear-thinning, *n* > 1 for shear-thickening, and *n* = 1 for ideally viscous flow behavior. Fitting to the Ostwald–de Waele model is appropriate where the measurement data are entirely within the shear-thinning regime across all the shear rates tested.

Cross model fluids behave similarly to those described by the Ostwald–de Waele model over a range of shear rates, but transition to regions of constant viscosity above and below this range. This model can be described according to Equation (2):

(2) $$\eta =\eta_{\infty }+\frac{\left(\eta_{0}-\eta_{\infty }\right)}{\left(1+{\left(\frac{ẏ}{ẏ\times \beta }\right)}^{n}\right)}$$

where *η_0_* is the zero-shear viscosity, *η_∞_* is the infinite-shear viscosity, and ẏ and *n* are fluid-specific parameters.

Herschel–Bulkley fluid relates the shear stress to the strain rate and can be described mathematically as described in Equation (3):

(3) $$\eta =\left(\frac{\tau_{0}}{ẏ}\right)+\left(k\;x\;{ẏ}^{n-1}\right)$$

where *τ*_0_ is the yield stress, *k* is the consistency factor, and *n* is the flow index.

The material follows a shear-thinning flow behavior. The Bingham plastic model can best reflect such flow and the model can be described according to Equation (4) [14].

(4) $$\eta =\eta_{\rho }+\frac{\tau_{0}}{ẏ}$$

where *η_ρ_* is the plastic viscosity and *τ*_0_ stands for the yield stress.

Finally, the Casson model is also used to model flow curves showing a yield point, reported as Equation (5):

(5) $$\eta =\sqrt[{n}]{{\left(\frac{\tau_{0}}{ẏ}\right)}^{n}+{\left(\eta_{\rho }\right)}^{n}}$$

where *τ*_0_ is Casson yield point and *η_ρ_* is the Casson viscosity [41,59,74,75,76].

Regarding Experiment 5 from DoE, as suggested per the desirability results, and considering the R^2^ values, the Herschel–Bulkley R^2^ = 0.9534) model provided the best ability for predicting the shear flow behavior (Table 10). The data retrieved from the model are consistent with the shear-thinning flow behavior exhibited when a stress larger than the yield stress is reached. Note that the accurate determination of *τ*_0_ is dependent on both the rheological method and the model function used, being a parameter that highly impacts the spreadability of topical dosage forms and sensory attributes [41].

Thixotropic profile

Pseudoplastic systems can present a phenomenon called thixotropy, as is the case obtained in this work (Figure 6B), because even with the shear velocity removed, the system tends to regain the initial structure in such a way that the ascending and descending curves of the rheogram are displaced, resulting in a hysteresis area. The thixotropy is directly proportional to the hysteresis area; therefore, the larger the hysteresis area, the greater the formulation thixotropy [66,77]. The goal for the thixotropic method was to minimize the relative thixotropic area response (S_R_—Pa·s). In other words, the main target was to develop a method that enabled a more rapid and complete regeneration of the formulation [59].

Figure 4D displays the coefficient values attained for the S_R_. The high-magnitude interaction of coefficient *β*_13_ (peltier temperature and sample application) suggests an increase in this CAA. Experiment 1 shows a larger area of the thixotropic loop. Therefore, it can be expected that the formulation in these experimental conditions slowly recovers after the removal of the shear rate or stress [78,79]. Regarding the peltier temperature at 25 °C, the viscosity rises and slows extrusion. However, since all chemical processes slow at low temperatures, thixotropic recovery also slows. The desirability profiler suggests performing the thixotropic analysis at 32 °C for peltier temperature, thirty minutes for sample rest time, and with a spatula as the sample application mode (Figure S2).

##### Creep Recovery

Figure 6E illustrates the typical creep recovery behavior of a viscoelastic material. The creep curve with an upward curvature indicates that the structure breaks down quickly under the influence of the shear stress and a viscosity reduction should occur [14,80]. The creep recovery test is used to analyze the viscoelastic behavior by performing two shear stress steps. The goal for the creep recovery test was to maximize the responses creep equilibrium compliance (J_e_, Pa^−1^) and the elastic reformation (γ_e_, %). The shear compliance is the reciprocal value of the shear modulus which can be foreseen as “rigidity” [81,82]. The elastic reformation indicates the elastic portion of the viscoelastic behavior. Creep tests essentially provide information at low stress over long periods of time (equivalent to low frequencies), but at high frequencies, it could be unavoidably inaccurate [14]. The peltier temperature (*β*_1_) regards the main CMV that statistically impacts both equilibrium compliance and elastic reformation (Figure 5E,F). The desirability profiler suggests performing the creep recovery analysis at 32 °C for peltier temperature, thirty minutes for sample rest time, and sample application using a syringe (Figure S2).

##### Oscillatory Measurements

The oscillatory measurements are given by the elastic distribution, termed as the storage modulus (G′), since it represents the storage of energy and the viscous contribution, termed as the loss modulus (G″), since it represents energy loss. Oscillatory method DoE studies aimed to maximize the responses for amplitude (LVR region, yield point, flow point) and frequency sweep (storage modulus (G′—Pa), loss modulus (G″—Pa). These tests can be further divided into amplitude sweep tests and frequency sweep tests.

Amplitude sweep

Amplitude sweep is an important test to determine the linear viscoelastic region (LVR). The LVR regards the *plateau* in which the microstructure of the sample remains undisrupted. In other words, it represents the plateau where the sample maintains both G′ and G″ despite changes in the shear stress (Figure 6C) [59,83]. This is a critical input parameter for subsequent frequency-based measurements. Considering the coefficient magnitude, the main method parameter impacting the LVR response regards the peltier temperature. At lower temperatures, most of the LVR response is higher. In line with this observation, the interaction between the temperature and the sample rest time also proved to have a statistically significant effect on this CAA, with lower temperatures and lower rest times, yielding higher LVR values [84,85,86]. At higher temperatures, material components have more thermal energy and, hence, a lower stress input is required to initiate flow. Magnitude of the interaction coefficient *β*_12_ suggests that the existence of interactions between the factors for yield point response (Figure 5H). Consequently, yield point (*τ*_0.OSC_) tends to decrease with increasing temperature so long as there is no thermally induced structural enhancement at elevated temperature. For the flow stress, the *β*_2_ coefficient indicates a negative impact of sample rest time on this response.

The desirability profiler suggests performing the amplitude sweep analysis at 25 °C for peltier temperature, zero minutes for sample rest time, and sample application using a spatula (Figure S2).

Frequency sweep

Frequency sweep measurements enable the determination of the viscoelastic properties of a sample as a function of timescale [59,84]. After the LVR has been defined by amplitude sweep, its structure can be further characterized using frequency sweep analysis. The considered outputs in this test were the storage modulus (G′—Pa) and the loss modulus (G″—Pa) [87].

Factorial design results showed that a decrease in the peltier temperature increased both CAAs. However, the sample rest time and the application method also proved to be significant. The response is directly proportional to an increase in rest time and spatula application, enabling higher G′ and G″ values (Figure 4J,K). Figure 6C,D shows what could be classified as a well-structured system. In this case, particles are strongly associated; the G′ is greater than the G″ and both are almost independent of frequency (Figure 6D). The frequency sweep curve gives a good rheological description of how the product behaves during storage and application [88].

The desirability profiler suggests performing the frequency sweep analysis at 25 °C for peltier temperature, thirty minutes for sample rest time, and sample application using a syringe (Figure S2).

#### 3.2.4. Risk Assessment Update

After performing the optimization studies, the risk assessment analysis was updated in order to capture the reduced level of risk, based on our improved method understanding (please see Table 11). The updated analysis was used to assess high risk items and the activities underway in order to provide corrective actions. According to the results, the main failure modes, including sample application mode, sample rest time, and peltier temperature control, are still at the top of the ranking, and could be the main critical method variables that lead to incorrect data analysis.

FMECA was also most useful to define job considerations, quality data points, preventive method actions, and activities necessary to minimize failure risk. The updated levels demonstrate that these specific method variables should be carefully selected due to their significant impact on rheology CAAs.

The details of the risk assessment, considering how method failure might be detected, must be performed in the experiment. This approach may seem costly with light benefit. However, once the work is completed, the ongoing management of the risk-based FMECA is much simpler and the benefits include the development of stronger compliance defense in the method, both in terms of justification of the potential impact of an instrument failure on results and reduction of risks because the possibility of an undetected method failure has been significantly reduced [89,90].

#### 3.2.5. Optimal Operational Settings

The optimal operational settings (Table 12) were established following a multidimensional approach considering the relative impact of each CMV per rheology measurements based on method factors and settings.

### 3.3. Method Validation

According to the analytical guidelines, analytical method validation is an essential requirement to perform numerous assessments designed to verify that an analytical test system is suitable for its intended reason and is capable of providing robust and legitimate analytical data [49,91].

#### 3.3.1. Precision

Repeatability and intermediate precision of twelve replicates showed relative standard deviation values less than 15% for all measurements. The results are displayed in Table 13.

#### 3.3.2. Selectivity

To evaluate selectivity, i.e., the ability of the methods to accurately identify distinct formulations, three pairwise statistical comparisons were performed: (i) F1 vs. F2; (ii) F1 vs. F3; and (iii) F2 vs. F3. If the CAAs of each formulation present significant differences, the method is considered to be selective. The results summarized in Table 14 show that low *p*-values are obtained for most of the comparisons, indicating that there are significant differences among the formulations.

There were, however, no statistically significant differences found for zero-shear viscosity and yield point between F2 vs. F3 (*p*-value = 0.9944 and 0.1535, respectively). Nevertheless, these two CAAs display significant differences between F1 vs. F2 and F1 vs. F3; therefore, punctual results do not undermine the overall selectivity results.

**Figure 7:**
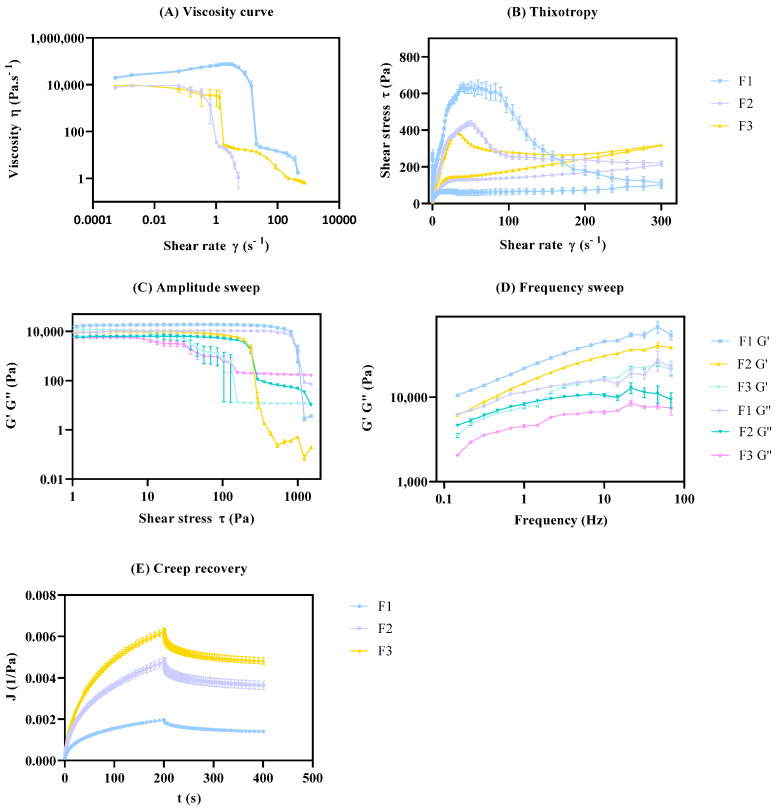
Rheology validation studies. Effect of glycerol monostearate (F2) and homogenization rate (F3) on rheology profile. Results report to n = 12 ± SEM. (**A**) Viscosity curve; (**B**) Thixotropic; (**C**) Amplitude sweep; (**D**) Frequency sweep; (**E**) Creep recovery.

From the data analysis (Figure 7), the formulation with distinct glycerol monostearate content (F2) and distinct manufacturing conditions (F3) displays significant differences in the cream microstructure, when compared to the nominal formulation (F1).

Please note that the evaluation of the method robustness could also have been performed; however, change in these CMVs cannot be equated due to their significant impact over the selected CAAs.

### 3.4. Summing-Up

❖ A detailed rheological characterization of a clobetasol propionate 0.5 mg/g cream formulation provided information on the product’s aesthetic properties, patient compliance, and overall quality profile;

❖ The AQbD concept, including a risk assessment to rank the impact of CMVs over CAAs, was considered in experimental design and the rheology measurement methods for the acquisition of a suitable rheology profile;

❖ A broad range of endpoints was considered throughout a rheology profile analysis, namely zero-shear viscosity, infinite-shear viscosity, rotational yield point, relative thixotropic area, creep equilibrium compliance, response elastic reformation, LVR *plateau*, oscillatory yield point, flow point, storage modulus, and loss modulus;

❖ Overall procedures and respective acceptance criteria regarding regulatory validation components such as equipment verification, precision, and discriminatory power were herein proposed;

❖ In view of the applicability and overall importance of rheology, this paper summarizes a practical standardization of procedures for the foundational development and validation of this method.

## 4. Bridging Compendial Testing with Rheometry-Based Approach

After rheology method development using an AQbD approach and validation, the rheology method’s applicability was determined by investigating the rheology profile of three batches of a commercially available clobetasol propionate 0.5 mg/g cream. The main objective of this analysis was to establish a quality control (QC) method for the rheological assessment of this specific topical product.

In addition to the RSD % (RSD < 15%) evaluation for each CAA, between the replicates, a range for acceptance criteria was established using the precision results to calculate the upper and lower control limits for each CAA. The low and high limits were estimated as follows: CAA average ± (standard deviation × 3) [92]. According to Table 15, the majority of the CAAs displayed compliant results concerning rotational, creep recovery, and oscillatory measurements, thus reinforcing the suitability of the proposed methods. Nevertheless, two samples (2 and 3) presented non-compliant results. Even though all the CAAs proved compliant with the previously established criteria (RSD < 15%), which are in agreement with FDA guidelines, a critical evaluation should be made on the specification range, bearing in mind the intrinsic variability associated with these dosage forms.

In quality control routine, viscometers are commonly employed to measure the viscosity of semisolid formulations during manufacture. Furthermore, the viscosity determination, by means of a rotational viscometer, is one of the analyses that are commonly presented in a Certificate of Analysis.

Typically, a viscometer employs a mechanical bearing that limits the speed and torque capabilities of the instrument, whereas a rheometer uses a low-friction air bearing. This means a viscometer can be a solution for material process or production tests that require simple flow measurements on Newtonian materials (where viscosity is independent of shear rate). However, the acquisition of a complete rheology profile warrants the determination of a better characterization of flow and deformation, as it measures the viscosity of the sample over a predetermined shear rate/shear stress range.

**Figure 8:**
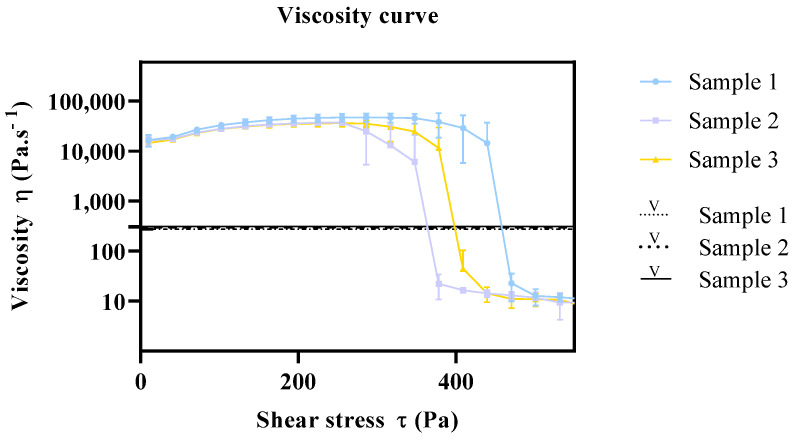
Viscosity relationship of rheometry and viscometer. Results report to n = 6 ± SEM. Key: V: viscometer samples.

In this context, the viscosity of the same commercial samples was also analyzed by a conventional viscometer, in order to establish a relationship with the viscosity results obtained with the rheometer. Six measurements were performed, and average viscosity values were calculated. The viscosity results for samples 1, 2, and 3 were 309 Pa·s^−1^ (SD 0.73), 300 Pa·s^−1^ (SD 1.11), and 274 Pa·s^−1^ (SD 0.88), respectively. Figure 8 depicts the viscosity curve from the rheometer and the relative viscosity from the viscometer. As it can be seen, the relative viscosity measurements comprise the shear-thinning range of the viscosity curve, which does not resemble the viscosity of the product in the container nor the viscosity of the sample upon application. This highlights the relevance of rheology method development, which provides a comprehensive knowledge on the viscosity, flow behavior, and yield point of the product under development, ultimately yielding a more efficient optimization of the product manufacturing process.

## 5. Conclusions

An AQbD-based rheology method for rotational, creep recovery, and oscillatory measurements was timely developed and validated under the umbrella of a comprehensive regulatory framework. Following risk assessment and factorial design tools, the impact of peltier temperature, sample rest time, and sample application on the responses stemming from rotational, creep recovery, and oscillatory measurements was estimated. DoE findings led to the definition of the best settings to describe the precision and selectivity of the rheological method, underlying “the right conditions at the first time” approach. Ultimately, the method was successfully applied to the analysis of the commercial products and could be used for routine quality control in pharmaceutical environments.

## Figures and Tables

**Table 1:** Qualitative composition of the clobetasol propionate cream formulation.

Components	Function
Clobetasol propionate	Active pharmaceutical ingredient
Chlorocresol	Preservative
Glyceryl monostearate	Emulsifying agent, emollient
Cetostearyl alcohol	Emulsifying agent, emollient
Citric acid	pH regulator
Sodium citrate	pH regulator
Propylene glycol	Co-solvent
Beeswax	Stabilizer agent
Purified water	Solvent

Note: for confidential reasons, the quantitative composition cannot be disclosed.

**Table 2:** Formulations addressed for DoE and method validation studies.

Formulation	Description	Studies Used
F1	Glycerol monostearate: nominal % Homogenization rate: nominal speed	DoE/Method validation: Precision
F2	Glycerol monostearate: lower % Homogenization rate: nominal speed	Method validation: Selectivity
F3	Glycerol monostearate: nominal % Homogenization rate: lower speed	Method validation: Selectivity

**Table 3:** Viscosity values from a Newtonian reference standard. Results report to n = 3.

Temperature	Viscosity *η* (Pa·s^−1^)
Standard 25 °C	Mean = 12.50 RSD = 6.36%
Standard 32 °C	Mean = 11.55 RSD = 6.29%

**Table 4:** Analytical target profile elements considered for the optimization of the rheology method for a 0.5 mg/g clobetasol propionate cream formulation.

ATP Element		Target/Objective (s)	Justification	Specification
Sample type (What/Where should be measured?)		Semisolid complex dosage forms: o/w cream formulations	Development of an analytical method that seeks to characterize the rheological behavior of a semisolid complex pharmaceutical dosage form.	N.A.
Product type (When should it be measured?)		Product development stages; Stability studies; Marketed products	The pharmaceutical product must display a viscosity profile that conforms to skin application.	N.A.
Method application		Characterization of a semisolid dosage form and validation of the developed method	The rheological properties such as viscosity and thixotropy of semisolid dosage forms need to be inspected, as they may influence drug delivery as well as impact patient adherence to treatment.	N.A.
Analytical method		Rheological analysis	Taking into account regulatory recommendations, the rheology analysis should comprehend the rotational profile (a complete flow curve, thixotropic relative area), creep test, as well as the oscillatory profile (frequency sweep and amplitude sweep) measurements.	N.A.
Equipment		Rotational rheometer equipped with a peltier system as a temperature control unit	A rheometer shears the sample between an upper rotating cone/plate and a lower fixed plate. The shear stress applied comes directly from the torque. The induced shear promotes the formation of horizontal layers of the sample. Due to this configuration, the rheometer requires a small amount of sample, and at the same time, it enables a rigorous control of the applied shear rate. From the rheological analysis, it is possible to retrieve a vast range of rheology endpoints. The temperature of the rheological tests needs to be controlled; furthermore, the minimization of sample volatilization during the analysis should also be actively pursued.	N.A.
Rheology critical analytical attributes (CAAs)		Zero-shear viscosity (*η*_0_, Pa·s^−1^); Infinite-shear viscosity (*η*_∞_, Pa·s^−1^); Yield point (*τ*_0.ROT_, Pa); Relative thixotropic area (S_R_, Pa·s); Creep equilibrium compliance (J_e_, Pa^−1^); Response elastic reformation (γ_e_, %); LVR *plateau* (LVR, Pa); Yield point (*τ*_0.OSC_, Pa); Flow point (*τ*_f_, Pa); Storage modulus (G′, Pa); Loss modulus (G″, Pa).	The relevance of each CAA is detailed in the following sections.	These CAAs should reflect the maximization of the rheology profile, except for the S_R_ where a more rapid recuperation of the formulation structure aimed to lower S_R_.
Method validation parameters	Discriminatory power Selectivity	Capacity of the method to distinguish between different formulations	A solid documentation of method discriminatory ability is progressively being demanded by regulatory authorities.	Selectivity: The differences between formulations should be statistically significant (ANOVA and Tukey test).
	Precision	Capacity to reproduce the operation over a short period of time	Degree of agreement among individual test results when an analytical method is used repeatedly on multiple samplings of a homogeneous sample.	An RSD less than 15% is considered acceptable from the mean CAAs [50].

**Table 5:** FMECA criteria to set up analysis scores.

	Score	Criteria
Severity (S)	1 (very low)	No impact to method quality
	2 (low)	No impact to method quality
	3 (average)	Noticeable impact to method quality, but can be recovered by reprocessing
	4 (high)	Definite impact to method quality that may require attention
	5 (very high)	Very severe effect, requires particular attention
Occurrence (O)	1 (unlikely)	Negligible risk which does not require attention
	2 (remote)	Failure only seen once or twice
	3 (occasional)	Failure potential has been noted
	4 (moderate)	Moderate-probability occurrence
	5 (likely)	Highly severe effect which requires utmost attention
Detection (D)	1 (very low)	Easily detectable; negligible risk which does not require attention
	2 (low)	Good detectability: possesses minor risk which can be corrected
	3 (average)	Detectable; risk which can be corrected
	4 (high)	Not easily detectable; risk requires attention
	5 (very high)	Very difficult to detect; risk which requires immediate attention

**Table 6:** Critical analytical attributes considered for the rheological tests.

Test	Response (CAAs)
Viscosity curve	Zero-shear viscosity (*η*_0_, Pa·s) Infinite-shear viscosity (*η*_∞_, Pa·s) Yield point (*τ*_0.ROT_, Pa)
Thixotropy	Relative thixotropic area (S_R_, Pa·s)
Creep recovery	Creep equilibrium compliance (J_e_, Pa^−1^); Response elastic reformation (γ_e_, %)
Amplitude sweep	LVR *plateau* (LVR, Pa) Yield point (*^τ^*_0.OSC_, Pa) Flow point (*^τ^*_f_, Pa)
Frequency sweep	Storage modulus (G′, Pa) Loss modulus (G″, Pa)

**Table 7:** Failure modes studied in FMECA for rheology method.

Failure Mode	Failure Cause	Failure Effect	Severity	Occurrence	Detection	RPN Score	Recommended Action (s)
CP geometry	Failure to choose geometry	The shear rate is not constant over the whole radius in the measuring gap	4	3	3	36	PP geometry can be employed. Effective shear rate varies across a parallel plate.
PP geometry		Viscosity values contain an intrinsic error	4	3	3	36	CP geometry can be employed. The diameter of the geometry has to be chosen in relation to the sample’s viscosity.
Sample application	Skills and training of the analyst	Sample should be carefully placed in plate	5	5	3	75	Standardize sample application. i.e., apply directly with a syringe or spatula in plate.
Sample amount	Do not apply the sample at the center of the plate	Under-filled sample can cause lower torque contribution; Over-filled sample can cause additional stress from drag along the edges	4	3	3	36	Standardize the quantity of the sample.
Sample rest time	Lack of scientific knowledge	Absence in sample rest time	5	4	3	60	Standardize when to start the analysis, after sample application.
Gap	Lack of equipment specifications knowledge	As gap height decreases, shear rate increases. Small gaps give high shear rates	5	3	3	45	Choose a suitable measuring gap.
Zero gap		Any error of the zero gap will automatically lead to an increased error of the test results due to a wrong gap size during the test	2	3	1	6	Zero gap should always be employed when the geometry is removed. Furthermore, to avoid any error due to thermal expansion, the zero gap has to be determined at the method’s working temperature.
Peltier temperature	Lack of scientific knowledge	Viscosity and other rheological parameters strongly depend on temperature	5	5	5	125	A system with peltier plate temperature should be employed.

**Table 8:** Critical method variables (CMVs) for experimental design and respective codification to assess the behavior of each condition on rotational and oscillatory measurements.

Factors (CMVs)	Role	Levels	
Sample application mode	Categorical	Syringe (−)	Spatula (+)
*Peltier* temperature control (°C)	Continuous	25 (−)	32 (+)
Sample rest time (min)	Continuous	0 (−)	30 (+)

**Table 9:** Design matrix used for optimization of rotational and oscillatory measurements.

Experiment	Peltier Temperature Control (°C)	Sample Rest Time (min)	Sample Application Mode
1	25	0	Spatula
2	25	0	Syringe
3	25	30	Spatula
4	25	30	Syringe
5	32	0	Spatula
6	32	0	Syringe
7	32	30	Spatula
8	32	30	Syringe

**Table 10:** Regression parameters resulting from the different rheological models fitting to the acquired rheological data (experiment 5).

Rheological Model	Rheology Equation	Estimated Parameters	R^2^ Values
Bingham	$\eta =1.05^{4}+\frac{14.047}{ẏ}$	*η_ρ_*: plastic viscosity; *τ*_0_: yield stress.	0.4572
Cross	$\eta =40.16+\frac{\left(4.59^{4}-40.16\right)}{\left(1+{\left(\frac{ẏ}{ẏ\times 1.80}\right)}^{5.313}\right)}$	*η*_0_: zero-shear viscosity; *η_∞_*: infinite-shear viscosity; *ẏ* and *n*: fluid-specific parameters.	0.9321
Herschel–Buckley	$\eta =\left(\frac{-55.18}{ẏ}\right)+\left(3123.14x{ẏ}^{0.5217-1}\right)$	*τ*_0_: yield stress; *k*: consistency factor; *n*: flow index.	0.9534
Ostwald–de Waele	$\eta =1.07^{4}x{ẏ}^{0.7774-1}$	*η*: viscosity (Pa·s^−1^); *k*: flow coefficient; *n*: flow index.	0.8286
Casson	$\eta =\sqrt[{0.5}]{{\left(\frac{8.066}{ẏ}\right)}^{0.5}+{\left(7743\right)}^{0.5}}$	*τ*_0_: Casson yield point; *η_ρ_*: Casson viscosity.	0.6213

**Table 11:** Updated FMECA after rheology method optimization.

Failure Mode	Failure Cause	Failure Effect	Severity	Occurrence	Detection	RPN * Score	Recommended Action (s)
CP geometry	Failure to choose geometry	The shear rate is not constant over the whole radius in the measuring gap	3	2	1	6	PP geometry can be employed. Effective shear rate varies across a parallel plate.
PP geometry		Viscosity values contain an intrinsic error	3	2	1	6	CP geometry can be employed. The diameter of the geometry has to be chosen in relation to the sample’s viscosity.
Sample application	Skills and training of the analyst	Sample should be carefully placed in plate	5	3	3	45	Standardize the sample application. directly apply the sample with the syringe or spatula in plate.
Sample amount	Do not apply the sample at the center of the plate	Under-filled sample can cause lower torque contribution; Over-filled sample can cause additional stress from drag along the edges	1	2	3	6	Standardize the sample quantity.
Sample rest time	Lack of scientific knowledge	Absence in sample rest time	5	3	3	45	Standardize when starting the analysis, after the sample application in plate.
Gap	Lack of equipment specifications knowledge	As gap height decreases, shear rate increases. Small gaps give high shear rates	3	1	1	3	Choose a suitable measuring gap.
Zero gap		Any error of the zero gap will automatically lead to an increased error of the test results due to a wrong gap size during the test	2	3	1	6	Zero gap should always be employed when the geometry is removed. Furthermore, to avoid any error due the thermal expansion, the zero gap has to be determined at the method’s working temperature.
Peltier temperature	Lack of scientific knowledge	Viscosity and other rheological parameters strongly depend on temperature	5	3	3	45	A system with peltier plate temperature could be employed.

* Risk Priority Number (RPN = S × O × D).

**Table 12:** CMVs’ effect on the meaningful rheology method performance CAAs.

CAAs	Peltier Temperature Control (°C)	Sample Application		Sample Rest Time (min)		Desirability (D)
		Syringe	Spatula	0	30	
Zero-shear viscosity (*η*_0_—Pas·s); Infinite-shear viscosity (*η*_∞_—Pa.s); Yield point (*τ*_0.ROT_—Pa)	25		X	X		0.3328
	32		X	X		0.5014
Relative thixotropic area (S_R_—Pa·s)	25		X		X	0.3356
	32		X		X	0.8683
Equilibrium compliance (Je, Pa^−1^); Elastic reformation (γe, %)	25	X			X	0.1508
	32	X			X	0.8014
Viscoelastic region LVR *plateau* (LVR—Pa); Yield point (*τ*_0.OSC_—Pa); Flow stress, flow point (*τ*_f_—Pa)	25		X	X		0.6809
	32		X	X		0.47175
Storage modulus (G′—Pa); Loss modulus (G″—Pa)	25	X			X	0.8335
	32	X			X	0.3425

Key: X, Best performance setting at a fixed temperature of 25 or 32 °C.

**Table 13:** Repeatability and intermediate precision results from rotational and oscillatory measurements. Results report to a n = 12 ± SEM.

CAAs	Repeatability		Intermediate Precision	
	Mean ± SEM	RSD (%)	Mean ± SEM	RSD (%)
Zero-shear viscosity (*η*_0_—Pas.s)	37,246 ± 1027	9.55	38,181 ± 870	11.16
Infinite-shear viscosity (*η*_∞_—Pa.s)	15.2 ± 0.5	9.01	15.5 ± 0.4	11.65
Yield point (*τ*_0_._ROT_—Pa)	369 ± 11	10.33	382 ± 10	13.24
Relative thixotropic area (S_R_—Pa/s)	76,863 ± 3143	14.16	76,886 ± 2117	13.49
Equilibrium compliance (J_e_—Pa^−1^)	0.00105 ± 2.96^−5^	9.75	0.00108 ± 2.19^−5^	9.91
Elastic reformation (γ_e_—%)	5.3 ± 0.1	9.77	5.4 ± 0.1	9.92
Viscoelastic region LVR *plateau* (LVR—Pa)	17,906 ± 723	13.99	17,114 ± 455	13.02
Yield point (*τ*_0.OSC_—Pa)	446 ± 10	7.97	441 ± 7	7.27
Flow stress, flow point (*τ*_f_—Pa)	897 ± 30	11.53	888 ± 19	10.38
Storage modulus (G′—Pa)	21,980 ± 491	7.73	22,269 ± 415	9.14
Loss modulus (G″—Pa)	11,403 ± 205	6.22	11,384 ± 148	6.36

**Table 14:** Selectivity results from rotational and oscillatory measurements. Results report to n = 12 ± SEM.

CAAs	Formulations		
	F1 vs. F2	F1 vs. F3	F2 vs. F3
Zero-shear viscosity (*η*_0_—Pas·s)	Normal distribution? Yes Cl: [26,083 to 30,441] *p*-value: <0.0001	Normal distribution? Yes Cl: [25,993 to 30,352] *p*-value: <0.0001	Normal distribution? No Cl: [−2269 to 2090] *p*-value: 0.9944
Infinite-shear viscosity (*η*_∞_—Pa·s)	Normal distribution? Yes Cl: [1.072 to 4.428] *p*-value: 0.0009	Normal distribution? Yes Cl: [−7.812 to −4.457] *p*-value: <0.0001	Normal distribution? Yes Cl: [−10.56 to −7.207] *p*-value: <0.0001
Yield point (*τ*_0.ROT_—Pa)	Normal distribution? Yes Cl: [319.7 to 363.9] *p*-value: <0.0001	Normal distribution? Yes Cl: [302.6 to 346.7] *p*-value: <0.0001	Normal distribution? No Cl: [−39.22 to 4.948] *p*-value: 0.1535
Relative thixotropic area (S_R_—Pa·s)	Normal distribution? Yes Cl: [36,245 to 49,711] *p*-value: <0.0001	Normal distribution? Yes Cl: [48,161 to 61,627] *p*-value: <0.0001	Normal distribution? Yes Cl: [5183 to 18,649] *p*-value: 0.0004
Equilibrium compliance (J_e_—Pa^−1^)	Normal distribution? Yes Cl: [−0.001512 to −0.001012] *p*-value: <0.0001	Normal distribution? Yes Cl: [−0.002417 to −0.001917] *p*-value: <0.0001	Normal distribution? Yes Cl: [−0.001155 to −0.0006548] *p*-value: <0.0001
Elastic reformation (γ_e_—%)	Normal distribution? Yes Cl: [−7.562 to −5.061] *p*-value: <0.0001	Normal distribution? Yes Cl: [−12.08 to −9.583] *p*-value: <0.0001	Normal distribution? Yes Cl: [−5.773 to −3.272] *p*-value: <0.0001
Viscoelastic region LVR *plateau* (LVR—Pa)	Normal distribution? Yes Cl: [6761 to 9961] *p*-value: <0.0001	Normal distribution? Yes Cl: [4915 to 8115] *p*-value: <0.0001	Normal distribution? Yes Cl: [−3446 to 8115] *p*-value: 0.0209
Yield point (*τ*_0.OSC_—Pa)	Normal distribution? Yes Cl: [348 to 394] *p*-value: <0.0001	Normal distribution? Yes Cl: [305 to 352] *p*-value: <0.0001	Normal distribution? Yes Cl: [−66 to 20] *p*-value: 0.0002
Flow stress, flow point (*τ*_f_—Pa)	Normal distribution? Yes Cl: [604 to 728] *p*-value: <0.0001	Normal distribution? Yes Cl: [710 to 835] *p*-value: <0.0001	Normal distribution? Yes Cl: [45 to 169] *p*-value: 0.0005
Storage modulus (G′—Pa)	Normal distribution? Yes Cl: [5995 to 8704] *p*-value: <0.0001	Normal distribution? Yes Cl: [12,569 to 15,278] *p*-value: < 0.0001	Normal distribution? Yes Cl: [5220 to 7929] *p*-value: <0.0001
Loss modulus (G″—Pa)	Normal distribution? Yes Cl: [2427 to 3752] *p*-value: <0.0001	Normal distribution? Yes Cl: [6082 to 7407] *p*-value: <0.0001	Normal distribution? Yes Cl: [2992 to 4317] *p*-value: <0.0001

**Table 15:** Commercial clobetasol propionate 0.5 mg/g cream results. Results report to n = 6 ± SD.

CAAs	Acceptance Criteria	Sample 1			Sample 2			Sample 3		
		Mean ± SD	RSD (%)	Status	Mean ± SD	RSD (%)	Status	Mean ± SD	RSD (%)	Status
Zero-shear viscosity (*η*_0_—Pas·s)	25,398–50,964	32,935 ± 4298	13.05	C	28,060 ± 2208	7.87	C	30,989 ± 3685	11.89	C
Infinite-shear viscosity (*η*_∞_—Pa·s)	10–21	15 ± 2	14.41	C	13 ± 2	12.37	C	16 ± 2	12.42	C
Yield point (*τ*_0.ROT_—Pa)	230–534	327 ± 29	8.88	C	214 ± 31	14.29	NC	277 ± 29	10.36	C
Relative thixotropic area (S_R_—Pa·s)	45,773–107,999	59,997 ± 7775	12.96	C	69,210 ± 10,187	14.72	C	63,375 ± 7459	11.77	C
Equilibrium compliance (J_e_—Pa^−1^)	0.000762–0.001407	0.0011 ± 0.0001	12.50	C	0.0013 ± 0.0002	13.98	C	0.0016 ± 0.0002	10.47	NC
Elastic reformation (γe—%)	3.72–6.81	5.4 ± 0.7	12.49	C	6.7 ± 0.9	13.98	C	8.2 ± 0.9	10.46	NC
Viscoelastic region LVR *plateau* (LVR—Pa)	10,427–23,801	20,883 ± 1650	7.90	C	17,492 ± 1793	10.25	C	18,782 ± 923	4.91	C
Yield point (*τ*_0.OSC_—Pa)	345–537	464 ± 14	3.03	C	391 ± 34	8.69	C	455 ± 47	10.29	C
Flow stress, flow point (*τ*_f_—Pa)	612–1164	771 ± 89	11.52	C	543 ± 40	7.30	C	646 ± 64	9.98	C
Storage modulus (G′—Pa)	16,162–28,378	22,003 ± 3287	14.94	C	22,583 ± 1727	7.65	C	25,257 ± 1620	6.41	C
Loss modulus (G″—Pa)	9212–13,556	12,883 ± 1065	8.26	C	14,373 ± 1618	11.26	NC	15,895 ± 1604	10.09	NC

## Data Availability

The data is available upon request.

## Supplementary Materials

The following supporting information can be downloaded at: https://www.mdpi.com/article/10.3390/pharmaceutics15071810/s1, Figure S1. Actual plots displaying predictions of rotational and creep recovery measurement responses (CAAs) presenting a better goodness of fit from DoE. Results report to n = 3; Figure S2. Overall desirability for rotational measurement optimization, according to the target imposed per CMV from DoE. Results report to n = 3; Figure S3. Actual plots displaying predictions of oscillatory measurement responses (CAAs) presenting a better goodness of fit from DoE. Results report to n = 3; Figure S4. Overall desirability for oscillatory measurement optimization, according to the target imposed per CMV from DoE. Results report to n = 3; Table S1. DoE coefficient values for critical analytical attributes of rotational measurements and respective summary of fit of the selected critical method variable. * Statistically significant coefficients, as extracted from Student’s *t*-test. Results report to n = 3; Table S2. DoE coefficient values for critical analytical attributes of oscillatory measurements and respective summary of fit of the selected critical method variable. * Statistically significant coefficients, as extracted from Student’s *t*-test. Results report to n = 3; Table S3. ANOVA parameters for the characterization of the model fitting per CAA from DoE. Results report to n = 3.

## Abbreviations

AQbD	Analytical Quality by Design
FMECA	Failure mode, effects, and criticality analysis
FDA	Food and Drug Administration
EMA	European Medicines Agency
DoE	Design of experiments
CP	Clobetasol propionate
ATP	Analytical target profile
QTPP	Quality target product profile
CQA	Critical quality attribute
CAA	Critical analytical attribute
CMV	Critical method variable
RPN	Risk priority number
ANOVA	One-way analysis of variance
o/w	Oil-in-water
F1	Clobetasol propionate 0.5 mg/g cream formulation
F2	Clobetasol propionate 0.5 mg/g cream formulation manufactured with 5% *w/w* of glyceryl monostearate amount
F3	Clobetasol propionate 0.5 mg/g cream formulation with different manufactured process
CR	Controlled-rate
CS	Controlled-stress
γ	Shear rate
*τ*	Shear stress
J_e_	Creep compliance
γ_e_	Elastic reformation
LVR	Linear viscoelastic region
G″	Loss modulus
G′	Storage modulus
RSD	Relative standard deviation
S_R_	Thixotropic relative area
*τ*f	Flow point
*τ*_0.OSC_	Oscillatory yield point
*τ*_0.ROT_	Rotational yield point
ƞ_∞_	Infinite-shear viscosity
ƞ_0_	Zero-shear viscosity
CP	Cone–plate
PP	Plate–plate
