# Supplementary material for: Rheology of Complex Topical Formulations: An Analytical Quality by Design Approach to Method Optimization and Validation
Source: Pharmaceutics. 2023 Jun 24;15(7):1810. doi: 10.3390/pharmaceutics15071810 (PMC10385475; doi:10.3390/pharmaceutics15071810)
Supplement: Supplementary file 1 [file pharmaceutics-15-01810-s001.zip › pharmaceutics-2426122-supplementary.pdf]

Supplementary Material

Rheology of complex topical formulations: an Analytical Quality by Design Approach to Method Optimization and Validation

Lucas Chiarentin [1,2,3], Catarina Cardoso [2], Margarida Miranda [2,3,4,*] and Carla Vitorino [1,3,*]

1 Faculty of Pharmacy, University of Coimbra, Pólo das Ciências da Saúde, Azinhaga de Santa Comba, 3000-548 Coimbra, Portugal; lczenith@gmail.com
2 Laboratórios Basi Indústria Farmacêutica S.A., Parque Industrial Manuel Lourenço Ferreira, lote 15, 3450-232 Mortágua, Portugal; catarina.cardoso@basi.pt
3 Coimbra Chemistry Centre, Institute of Molecular Sciences—IMS, Department of Chemistry, University of Coimbra, 3000-535 Coimbra, Portugal
4 Egas Moniz Center for Interdisciplinary Research (CiiEM), Egas Moniz School of Health & Science, 2829-511 Caparica, Almada, Portugal
* Correspondence: margarida.miranda@basi.pt (M.M.); csvitorino@ff.uc.pt (C.V.); Tel.: +351-239-448-400 (C.V.)

List of Tables:

Table S1. Coefficient values for critical analyte attributes of rotational measurements and respective summary of fit of the selected critical method variable. *Statistical, significance coefficients, as extracted from Student's t-test analysis. 3
Table S2. Coefficient values for critical analyte attributes of oscillatory measurements and respective summary of fit of the selected critical method variable. *Statistical, significance coefficients, as extracted from Student's t-test analysis 4
Table S3. ANOVA parameters for the characterization of the model fitting per CAA. 9

List of Figures:

Figure S1. Actual by predicted plots of rotational and creep recovery measurements for the responses (CAAs) presenting a better goodness of fit....................5
Figure S2. Overall desirability for rotational measurements optimization, according to the target imposed per CMV. 6
Figure S3. Actual by predicted plots of oscillatory measurements for the responses (CAAs) presenting a better goodness of fit. 7
Figure S4. Overall desirability for oscillatory measurements optimization, according to the target imposed per CMV. 8

Table S1. DoE coefficient values for critical analyte attributes of rotational measurements and respective summary of fit of the selected critical method variable. *Statistical, significance coefficients, as extracted from Student's *t*-test analysis.

		**Zero-shear viscosity (η_0 – Pas.s)**		**Infinite-shear viscosity (η_∞ - Pa.s)**		**Yield point (τ0.ROT – Pa)**		**Relative thixotropic area (S_R – Pa/s)**		**Equilibrium compliance (Je Pa^{-1})**		**Elastic reformation (γe %)**	
	Term	Estimate	Prob>|t|	Estimate	Prob>|t|	Estimate	Prob>|t|	Estimate	Prob>|t|	Estimate	Prob>|t|	Estimate	Prob>|t|
β0	Intercept	27352.675	<.0001*	38.3952	<.0001*	89.3304	<.0001*	53707.5	<.0001*	0,0015102	<,0001*	7,5496667	<,0001*
β1	Peltier temperature (25 ºC,32 ºC)	1786.2771	0.0081*	-1.4091	0.1112	-6.5962	0.0704	-4500	0.0040*	0,0003397	<,0001*	1,6966667	<,0001*
β2	Sample rest time (0,30 min)	2005.7696	0.0037*	-1.6462	0.0662	-9.8221	0.0105*	-4598.333	0.0035*	3,6542e-6	0,8964	0,0193333	0,8906
β3	Sample application [Syringe]	-1155.161	0.0693	5.4359	<.0001*	-17.5879	<.0001*	-1225.833	0.3783	-2,918e-5	0,3060	-0,145	0,3097
β12	Peltier temperature*Sample rest time	-2010.151	0.0036*	-1.2010	0.1703	-3.9971	0.2583	-1289.167	0.3548	1,2929e-5	0,6460	0,0656667	0,6414
β13	Peltier temperature*Sample application [Syringe]	-3045.955	<.0001*	-1.6133	0.0713	12.2021	0.0024*	9615	<.0001*	0,0000686	0,0239*	0,3443333	0,0236*
β23	Sample rest time*Sample application [Syringe]	2760.2788	0.0002*	-1.7828	0.0485*	8.1963	0.0282*	-1391.667	0.3188	-2,429e-6	0,9310	-0,012833	0,9273
R^2		0.8301		0.7762		0.7744		0.8165		0.9031		0.9031	
R^2 Adj		0.7701		0.6972		0.6947		0.7518		0.8689		0.8683	
Root Mean Square Error		2918.804		4.1088		16.7422		6638.78		0.000135		0.6784	

Table S2. DoE coefficient values for critical analyte attributes of oscillatory measurements and respective summary of fit of the selected critical method variable. *Statistical, significance coefficients, as extracted from Student's *t*-test analysis. Results report to n=3.

		**Viscoelastic region LVR plateau (LVR - Pa)**		**Yield point ($\tau_{0.OSC}$ - Pa)**		**Flow stress, flow point (τ_f – Pa)**		**Storage modulus (G′ – Pa)**		**Loss modulus (G′′ – Pa)**	
	Term	Estimate	Prob>|t|	Estimate	Prob>|t|	Estimate	Prob>|t|	Estimate	Prob>|t|	Estimate	Prob>|t|
β0	Intercept	7616.5	<.0001*	318.1583	<.0001*	294.7542	<.0001*	10562.708	<.0001*	6518.4167	<.0001*
β1	Peltier temperature (25 °C,32 °C)	-2096.333	<.0001*	24.8667	<.0001*	-4.6792	0.6073	-1963.958	<.0001*	-789.5	<.0001*
β2	Sample rest time (0,30 min)	245.6667	0.3097	-27.7167	<.0001*	-26.5042	0.0087*	671.2917	<.0001*	384.1667	0.0027*
β3	Sample application [Syringe]	97.6667	0.6824	-7.7667	0.0761	-12.7125	0.1729	414.875	0.0002*	329.0833	0.0079*
β12	Peltier temperature*Sample rest time	-595	0.0213*	34.525	<.0001*	10.0458	0.2765	-0.375	0.9967	-48.75	0.6613
β13	Peltier temperature*Sample application[Syringe]	-275	0.2573	1.1916	0.7755	9.6875	0.2934	-126.7917	0.1688	-197.3333	0.0888
β23	Sample rest time*Sample application[Syringe]	177.6667	0.4592	1.8417	0.6599	-19.4041	0.0443*	-74.0417	0.4130	-37.1667	0.7381
R^2		0.8404		0.9019		0.5178		0.9714		0.8194	
R^2 Adj		0.7841		0.8673		0.3476		0.9614		0.7556	
Root Mean Square Error		1149.238		20.1433		43.7726		432.2245		535.6198	

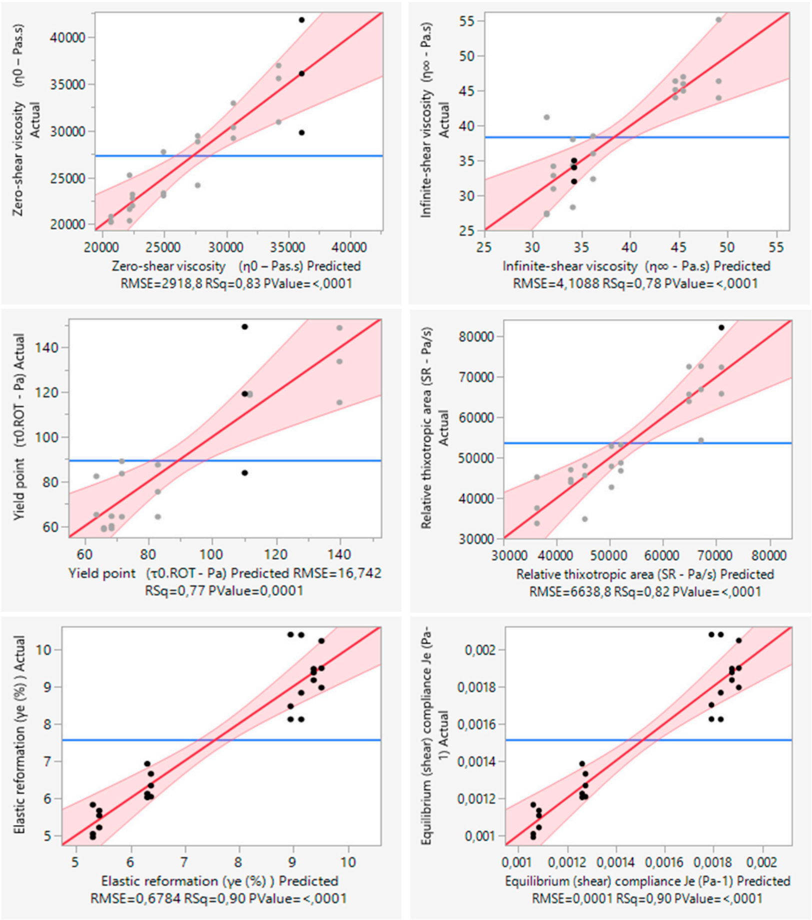

Figure S1. Actual by predicted plots of rotational and creep recovery measurements for the responses (CAAs) presenting a better goodness of fit from DoE. Results report to n=3.

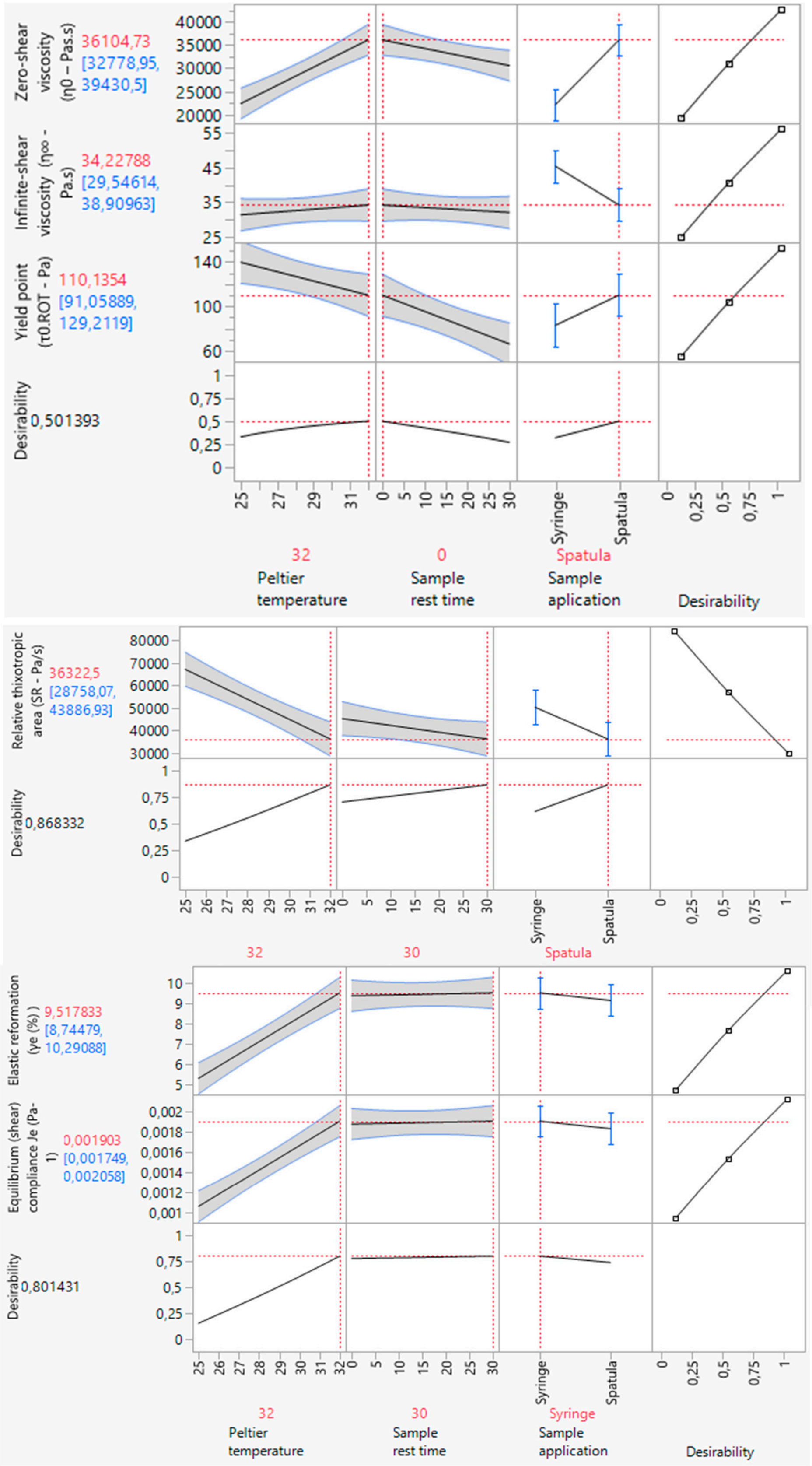

Figure S2. Overall desirability for rotational measurements optimization, according to the target imposed per CMV from DoE. Results report to n=3.

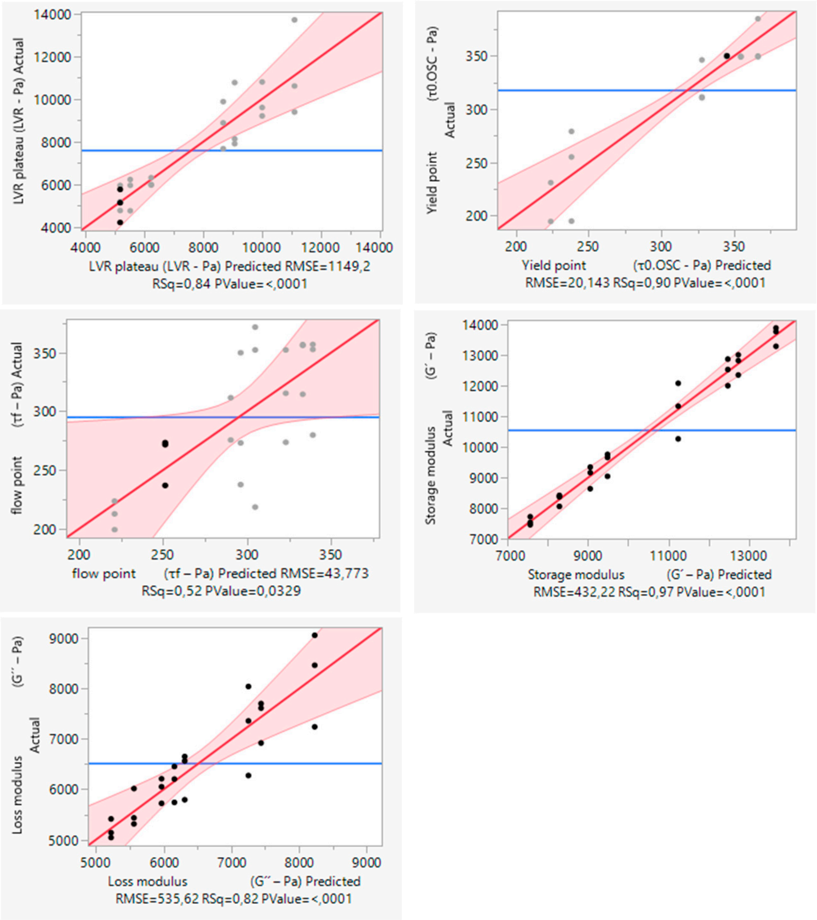

Figure S3. Actual by predicted plots of oscillatory measurements for the responses (CAAs) presenting a better goodness of fit from DoE. Results report to n=3.

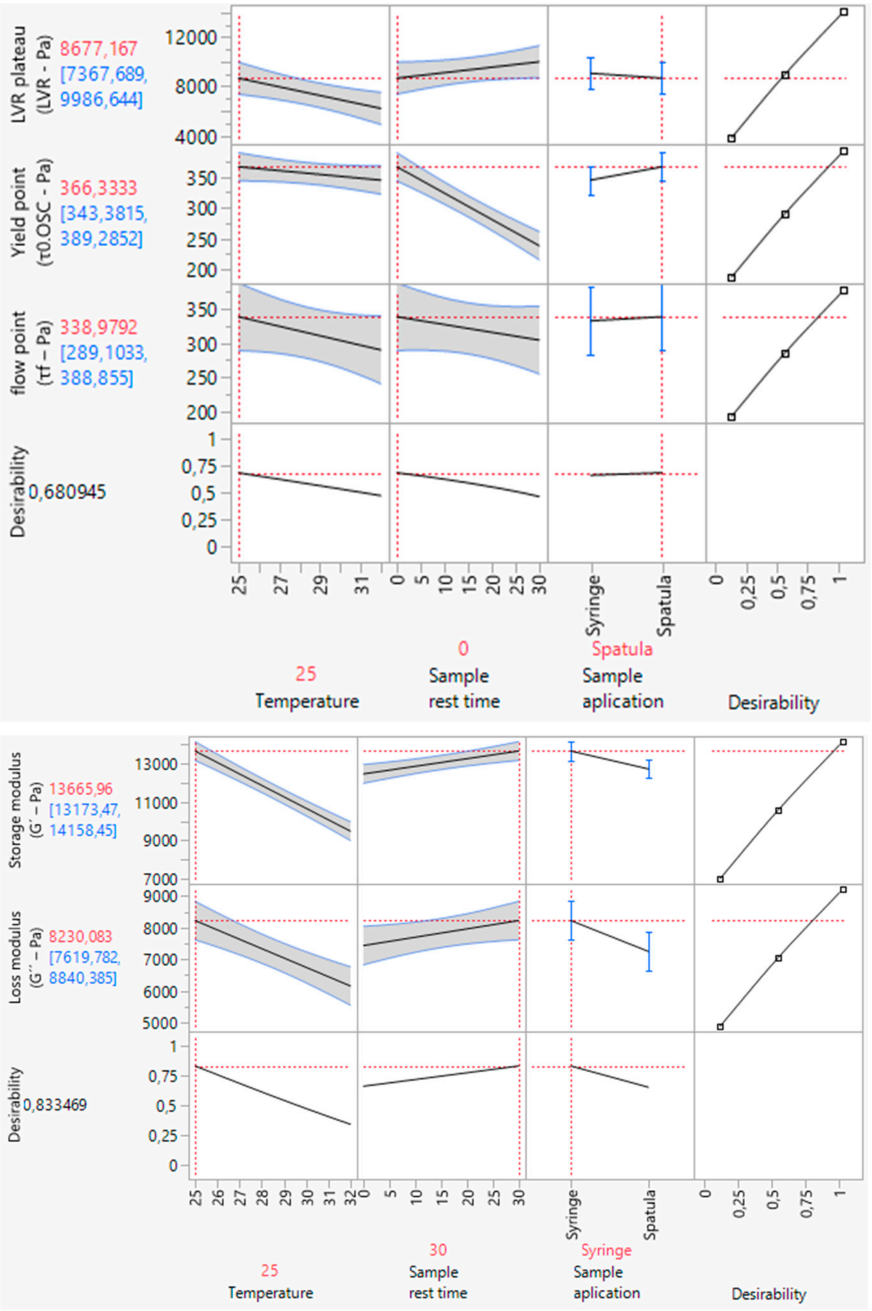

Figure S4. Overall desirability for oscillatory measurements optimization, according to the target imposed per CMV from DoE. Results report to n=3.

Table S3. ANOVA parameters for the characterization of the model fitting per CAA from DoE. Results report to n=3.

**Viscosity curve – Zero-shear viscosity**				
**Source**	**DF**	**Sum of Squares**	**Mean Square**	**F Ratio**
Model	6	707663668	117943945	13,8441
Error	17	144830081	8519416,5	**Prob > F**
C. Total	23	852493749		0,0001*
Lack Of Fit	1	1255604	1255604	0,1399
Pure Error	16	143574477	8973405	**Prob > F**
Total Error	17	144830081		0,7133
				**Max RSq**
				0,8316
**Viscosity curve – Infinite-shear viscosity**				
**Source**	**DF**	**Sum of Squares**	**Mean Square**	**F Ratio**
Model	6	995,2525	165,875	9,8252
Error	17	287,0047	16,883	**Prob > F**
C. Total	23	1282,2572		0,0001*
Lack Of Fit	1	7,55914	7,5591	0,4328
Pure Error	16	279,44557	17,4653	**Prob > F**
Total Error	17	287,00471		0,5200
				**Max RSq**
				0,7821
**Viscosity curve – Yield point**				
**Source**	**DF**	**Sum of Squares**	**Mean Square**	**F Ratio**
Model	6	16352,752	2725,46	9,7234
Error	17	4765,096	280,30	**Prob > F**
C. Total	23	21117,848		<,0001*
Lack Of Fit	1	1257,2985	1257,30	5,7349
Pure Error	16	3507,7970	219,24	**Prob > F**
Total Error	17	4765,0955		0,0292*
				**Max RSq**
				0,8339
**Thixotropic – Relative thixotropic area**				
**Source**	**DF**	**Sum of Squares**	**Mean Square**	**F Ratio**
Model	6	3334661967	555776994	12,6103
Error	17	749247883	44073405	**Prob > F**
C. Total	23	4083909850		<,0001*
Lack Of Fit	1	151704817	151704817	4,0621
Pure Error	16	597543067	37346442	**Prob > F**
Total Error	17	749247883		0,0610
				**Max RSq**
				0,8537
**Amplitude – LVR plateau**				
**Source**	**DF**	**Sum of Squares**	**Mean Square**	**F Ratio**
Model	6	118217275	19702879	14,9180
Error	17	22452729	1320748,8	**Prob > F**
C. Total	23	140670004		<,0001*
Lack Of Fit	1	425601	425601	0,3091
Pure Error	16	22027129	1376696	**Prob > F**
Total Error	17	22452729		0,5859
				**Max RSq**
				0,8434

Creep recovery – Elastic reformation (γ_e %)				
**Source**	**DF**	**Sum of Squares**	**Mean Square**	**F Ratio**
Model	6	72,554851	12,0925	26,2714
Error	17	7,824938	0,4603	**Prob > F**
C. Total	23	80,379789		<,0001*
Lack Of Fit	1	0,0374460	0,037446	0,0769
Pure Error	16	7,7874920	0,486718	**Prob > F**
Total Error	17	7,8249380		0,7850
				**Max RSq**
				0,9031

Creep recovery – Equilibrium compliance (J_e Pa^{-1})				
**Source**	**DF**	**Sum of Squares**	**Mean Square**	**F Ratio**
Model	6	2,907e-6	4,845e-7	26,4078
Error	17	3,11897e-7	1,8347e-8	**Prob > F**
C. Total	23	3,2189e-6		<,0001*
Lack Of Fit	1	1,60557e-9	1,6056e-9	0,0828
Pure Error	16	3,10292e-7	1,9393e-8	**Prob > F**
Total Error	17	3,11897e-7		0,7772
				**Max RSq**
				0,9036

Amplitude – Yield point				
**Source**	**DF**	**Sum of Squares**	**Mean Square**	**F Ratio**
Model	6	63448,158	10574,7	26,0620
Error	17	6897,780	405,8	**Prob > F**
C. Total	23	70345,938		<,0001*
Lack Of Fit	1	592,0267	592,027	1,5022
Pure Error	16	6305,7533	394,110	**Prob > F**
Total Error	17	6897,7800		0,2381
				**Max RSq**
				0,9104

Amplitude – Flow point				
**Source**	**DF**	**Sum of Squares**	**Mean Square**	**F Ratio**
Model	6	34974,269	5829,04	3,0422
Error	17	32572,690	1916,04	**Prob > F**
C. Total	23	67546,960		0,0329*
Lack Of Fit	1	2081,344	2081,34	1,0922
Pure Error	16	30491,347	1905,71	**Prob > F**
Total Error	17	32572,690		0,3115
				**Max RSq**
				0,5486

Frequency – Storage modulus				
**Source**	**DF**	**Sum of Squares**	**Mean Square**	**F Ratio**
Model	6	108034669	18005778	96,3814
Error	17	3175906	186818	**Prob > F**
C. Total	23	111210575		<,0001*
Lack Of Fit	1	852,0	852	0,0043
Pure Error	16	3175054,0	198441	**Prob > F**
Total Error	17	3175906,0		0,9486
				**Max RSq**
				0,9715

Frequency – Loss modulus				
**Source**	**DF**	**Sum of Squares**	**Mean Square**	**F Ratio**
Model	6	22125324	3687554	12,8536
Error	17	4877106	286889	**Prob > F**
C. Total	23	27002430		<,0001*
Lack Of Fit	1	15708,2	15708	0,0517

Pure Error	16	4861398,0	303837	**Prob > F**
Total Error	17	4877106,2		0,8230
				**Max RSq**
				0,8200
